# Effectiveness of sensor-based interventions in improving gait and balance performance in older adults: systematic review and meta-analysis of randomized controlled trials

**DOI:** 10.1186/s12984-024-01375-0

**Published:** 2024-05-28

**Authors:** Qian Mao, Jiaxin Zhang, Lisha Yu, Yang Zhao, Yan Luximon, Hailiang Wang

**Affiliations:** 1 School of Design, The Hong Kong Polytechnic University, Hong Kong, China; 2 School of System Design and Intelligent Manufacturing, Southern University of Science and Technology, Shenzhen, China; 3 School of Data Science, Lingnan University, Hong Kong, China; 4 School of Public Health (Shenzhen), Sun Yat-Sen University, Shenzhen, China

**Keywords:** Sensor-based technology, Older adults, Gait and balance, Physical exercise, Mobility rehabilitation, Biofeedback

## Abstract

**Background:**

Sensor-based interventions (SI) have been suggested as an alternative rehabilitation treatment to improve older adults’ functional performance. However, the effectiveness of different sensor technologies in improving gait and balance remains unclear and requires further investigation.

**Methods:**

Ten databases (Academic Search Premier; Cumulative Index to Nursing and Allied Health Literature, Complete; Cochrane Central Register of Controlled Trials; MEDLINE; PubMed; Web of Science; OpenDissertations; Open grey; ProQuest; and Grey literature report) were searched for relevant articles published up to December 20, 2022. Conventional functional assessments, including the Timed Up and Go (TUG) test, normal gait speed, Berg Balance Scale (BBS), 6-Minute Walk Test (6MWT), and Falling Efficacy Scale-International (FES-I), were used as the evaluation outcomes reflecting gait and balance performance. We first meta-analyzed the effectiveness of SI, which included optical sensors (OPTS), perception sensors (PCPS), and wearable sensors (WS), compared with control groups, which included non-treatment intervention (NTI) and traditional physical exercise intervention (TPEI). We further conducted sub-group analysis to compare the effectiveness of SI (OPTS, PCPS, and WS) with TPEI groups and compared each SI subtype with control (NTI and TPEI) and TPEI groups.

**Results:**

We scanned 6255 articles and performed meta-analyses of 58 selected trials (sample size = 2713). The results showed that SI groups were significantly more effective than control or TPEI groups (*p* < 0.000) in improving gait and balance performance. The subgroup meta-analyses between OPTS groups and TPEI groups revealed clear statistically significant differences in effectiveness for TUG test (mean difference (MD) = − 0.681 s; *p* < 0.000), normal gait speed (MD = 4.244 cm/s; *p* < 0.000), BBS (MD = 2.325; *p* = 0.001), 6MWT (MD = 25.166 m; *p* < 0.000), and FES-I scores (MD = − 2.036; *p* = 0.036). PCPS groups also presented statistically significant differences with TPEI groups in gait and balance assessments for normal gait speed (MD = 4.382 cm/s; *p* = 0.034), BBS (MD = 1.874; *p* < 0.000), 6MWT (MD = 21.904 m; *p* < 0.000), and FES-I scores (MD = − 1.161; *p* < 0.000), except for the TUG test (MD = − 0.226 s; *p* = 0.106). There were no statistically significant differences in TUG test (MD = − 1.255 s; *p* = 0.101) or normal gait speed (MD = 6.682 cm/s; *p* = 0.109) between WS groups and control groups.

**Conclusions:**

SI with biofeedback has a positive effect on gait and balance improvement among a mixed population of older adults. Specifically, OPTS and PCPS groups were statistically better than TPEI groups at improving gait and balance performance, whereas only the group comparison in BBS and 6MWT can reach the minimal clinically important difference. Moreover, WS groups showed no statistically or clinically significant positive effect on gait and balance improvement compared with control groups. More studies are recommended to verify the effectiveness of specific SI.

*Research registration* PROSPERO platform: CRD42022362817. Registered on 7/10/2022

**Supplementary Information:**

The online version contains supplementary material available at 10.1186/s12984-024-01375-0.

## Introduction

Aging has become a global issue [1]. According to the United Nations, the number of older adults is expected to reach 2.1 billion by 2050, and one in six individuals globally will be older than 60 years [2]. The population change has unavoidably presented new challenges to economics, housing and urban planning, and health and social care in some countries [3]. Moreover, negative physical and mental changes occur with aging [4, 5], resulting in an increased fall risk in older adults [6, 7]. Previous investigations have reported that approximately one in three people older than 65 years fall each year [2] and 55.8% of accidental deaths are attributable to falls [8]. In addition, 40% and 50% of fall-related injuries occur in individuals aged over 75 and 80 years, respectively [9, 10]. These fall-related consequences damage the physical health of older adults and reduce their confidence, activity independence, and social interactions [1].

Gait and balance deficits are considered the main risk factors for falls in older adults [11]. For example, a decrease in normal walking speed of 10 cm/s was linked with a 7% increase in the fall risk [12]. If the variability in stride or swing time increases by one standard deviation, the fall risk increases 5.3 and 2.2 times, respectively [8]. Poor postural stability and balance ability also contribute to increased fall risks [13, 14]. As diverse physiological systems collaborate to maintain balance ability, degradation in any one aspect can affect older adults’ balance ability [8]. For example, decreased muscle strength declines postural reaction and balance, which contributes to the risk of falls [15, 16]. Therefore, a series of interventions have been conducted in previous studies to improve the gait and balance of older adults [4, 8, 17].

Although gait and balance are considered independent clinical items, they are interactive and related to muscle strength and cognitive ability [1, 14]. Traditional physical exercises, including progressive resistance, strengthening, treadmill, balance, walking, and dual-task training, have been widely used and verified with positive effects in improving gait and balance performance [4, 17–19]. However, older adults were mostly required to perform these traditional physical exercises in healthcare institutions and under the supervision of physiotherapists [20]. The safety issues and timely information feedback limit the home use of these traditional physical exercises.

Recently, sensor-based technologies, such as exergaming, virtual reality, and wearable sensors, have been widely integrated with traditional physical exercises to improve the gait and balance performance of older adults [21–23]. Compared with traditional interventions, sensor-based technologies provide an interactive environment and immediate feedback about the performance of users [24, 25]. Moreover, the adherence and motivation of individuals in rehabilitation have been shown to be promoted by sensor-based technologies [20].

Sensor-based technologies can be divided into three types: optical sensor (OPTS), perception sensor (PCPS), and wearable sensor (WS) [2]. OPTS commonly collect users’ kinematic information and provide biofeedback of whole-body motion. Examples of OPTS include Kinect, infrared sensors, and cameras. PCPS, which include the Wii balance board and force platforms, are usually located on the ground or integrated into the environment to provide force biofeedback. WS, such as accelerometers and gyroscopes, are normally worn on the body to reflect users’ partial body motion.

With the wide application of sensor-based technologies in gait and balance rehabilitation, an increasing number of randomized controlled trials (RCTs) have been conducted to compare the effectiveness of sensor-based intervention (SI) with that of traditional physical exercise intervention (TPEI) in improving gait and balance performance in older adults. Several quantified measurements of gait and balance performance [2, 20], including the Timed Up and Go (TUG) test, normal gait speed, Berg Balance Scale (BBS), 6-Minute Walk Test (6MWT), and Falling Efficacy Scale-International (FES-I), have been introduced to assess the effectiveness of SI for older adults. Some randomized controlled trials have also assessed the effectiveness of different sensor technologies in improving certain gait and balance outcomes [19, 21, 23]. Meanwhile, several reviews and meta-analyses have investigated the role of sensor-based technologies in improving gait and balance performance in older adults [20, 25–28].

However, few studies have examined which type of sensor technology is more effective for gait and balance improvement. Different sensor-based technologies provide different biofeedback information on gait and balance interventions [25, 26]. To the best of our knowledge, the effectiveness of interventions using specific types of sensor technology in improving gait or balance is not yet understood. Furthermore, in most previous meta-analyses, the TPEI group and the non-treatment intervention (NTI) group have usually been included together in the control group, which may confound the effects of sensor-based technology with those of traditional physical exercise, making it more difficult to quantify the true effects of sensor-based technology.

Therefore, in this study, we attempted to fill these gaps by providing an updated comprehensive review on the topic and a meta-analysis of the effects of different types of sensor technologies on gait and balance performance in older adults. Specifically, we conducted subgroup analyses of three types of sensor-based technologies to examine the effectiveness of specific SI in improving gait and balance performance. In addition, to accurately compare the effectiveness of SI and TPEI groups, we further subdivided the control groups into TPEI groups and NTI groups throughout the study. Finally, we discussed in detail the results of each subgroup study and summarized the performance of different types of sensors in different conventional functional assessments.

## Methods

### Search strategy

This review was conducted based on the guidelines of Preferred Reporting Items for Systematic Reviews and Meta-Analyses [29] and was registered on the PROSPERO platform (CRD42022362817). Ten databases, namely, Academic Search Premier; Cumulative Index to Nursing and Allied Health Literature, Complete; Cochrane Central Register of Controlled Trials; MEDLINE; PubMed; Web of Science; OpenDissertations; Open grey; ProQuest; and Grey literature report, were searched for relevant articles published up to December 20, 2022. The search terms included: (wearable* OR sensor* OR acceler * OR gyro* OR magnetometer* OR camera* OR track* OR exergam* OR virtual reality OR VR OR augmented reality OR AR) AND (gait OR walk* OR balanc* OR postur* OR mobility) AND (old* OR elder* OR senior* OR aged OR geriatr* OR gerontol*) AND (train* OR program* OR exercise* OR intervention*) AND (random* OR randomized controlled trial* OR RCT*).

### Study selection

Studies were included if they: (a) were RCTs; (b) compared the use of sensor-based technology with non-use of the technology for gait and/or balance performance; (c) examined older adults with an average age > 60 years; (d) were published in peer-reviewed journals; and (e) were written in English. Review articles, case studies, commentary letters, and studies with only qualitative data analyses were excluded. The titles and abstracts of the articles identified in the initial search were screened to determine their relevance. The full text of potentially relevant articles was then reviewed for final inclusion. Reference lists of the included articles were also examined for any missed studies. Two authors (Q.M. and J.Z.) assessed the articles independently, and any discrepancies in study inclusion were solved by discussions with the third author (H.W.).

### Data extraction

The extracted data included: (1) the study characteristics, including first author, published year, and region; (2) the study design and participants, including sample size and groups in the RCTs and the gender distribution and age of participants; (3) the intervention strategies, including technologies used for the sensors, the therapies used for the intervention and control groups, and the treatment duration and frequency; and (4) outcomes with respect to gait and/or balance performance.

Specifically, we primarily analyzed gait and balance performance after the interventions and aimed to assess the differences between SI groups and control groups. Furthermore, we divided the sensor technologies into OPTS, PCPS, and WS. The control groups were also subdivided into TPEI groups and NTI groups. Therefore, we excluded SI in which more than two sensor technologies were used and included SI groups with single interventions if there were several SI in a trial. For example, if there were four groups including the SI, SI + TPEI, TPEI, and NTI groups in a trial, we only included the SI, TPEI, and NTI groups in the meta-analysis. The meta-analyses were performed only for gait and balance outcomes examined in at least three trials, which is the typical minimum standard [30]. Furthermore, we only included the outcomes analyzed for at least two sensor technologies.

### Data analysis

We assessed the effect size of each trial using the mean differences in outcomes, with 95% confidence intervals, based on suggestions provided in previous meta-analysis studies [31]. The heterogeneity of the studies was evaluated using the inconsistency test (I^2^). An I^2^ value less than 40% was considered to indicate low heterogeneity [32]. Meta-regression and subgroup analyses were used to determine the significant mediators responsible for the heterogeneity. The mediators included the sensor technology of SI groups, the intervention strategy of control groups, and participants’ age and health status. We used random-effects models to pool the effect sizes for trials with high levels of heterogeneity [32]. Egger’s regression tests were used to assess publication bias when more than 10 trials evaluated the outcomes, with *p* < 0.05 indicating the presence of publication bias [33]. Our analysis was performed using Comprehensive Meta-Analysis 3.0 (Biostat, Inc., Tampa, FL, USA) with significance levels predetermined at *p* < 0.05.

**Fig. 1:**
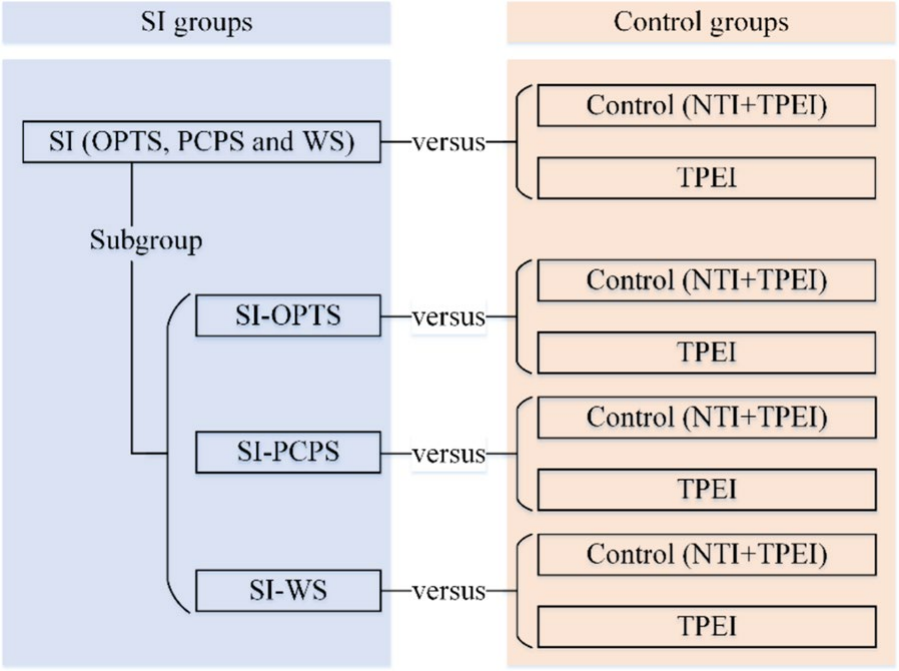
The strategy of meta-analyses in this study

For each outcome, we initially introduced the intervention characteristics of the included studies. We provided the total number of trials included in the meta-analysis, along with the sample sizes of the intervention (SI) groups and control groups. Additionally, we described the percentage of each sensor type used in the interventions. In terms of the meta-analyses, we first meta-analyzed the effectiveness of SI, including OPTS, PCPS, and WS, compared with control groups, including NTI and TPEI. We further conducted sub-group analysis to compare the effectiveness of SI (OPTS, PCPS, and WS) with TPEI groups, and each SI subtype was compared with control (NTI and TPEI) and TPEI groups. Figure 1 presents the strategy of the meta-analyses in this study.

### Risk of bias assessment

The revised Cochrane Risk-of-Bias tool was used independently by two authors (Q.M. and J.Z.) to assess the risk of bias in the included trials. The assessment indices of the tool consist of the randomization process, deviations from the intended interventions, missing outcome data, measurement of the outcomes, and selection of the reported results [34]. Each domain was classified into “low risk,” “some concerns,” or “high risk” based on the responses to the items, and an overall assessment was calculated based on the five domains.

### Quality of evidence assessment

The quality of evidence was classified as high, moderate, low, or very low according to the Grading of Recommendations Assessment, Development, and Evaluation (GRADE) system [35]. Two authors (Q.M. and J.Z.) assessed the overall rating of the quality of evidence based on the evaluation of the risk of bias, imprecision, inconsistency, indirectness, publication bias, effect size, dose response, and confounding factors [35].

## Results

### Study characteristics

**Fig. 2:**
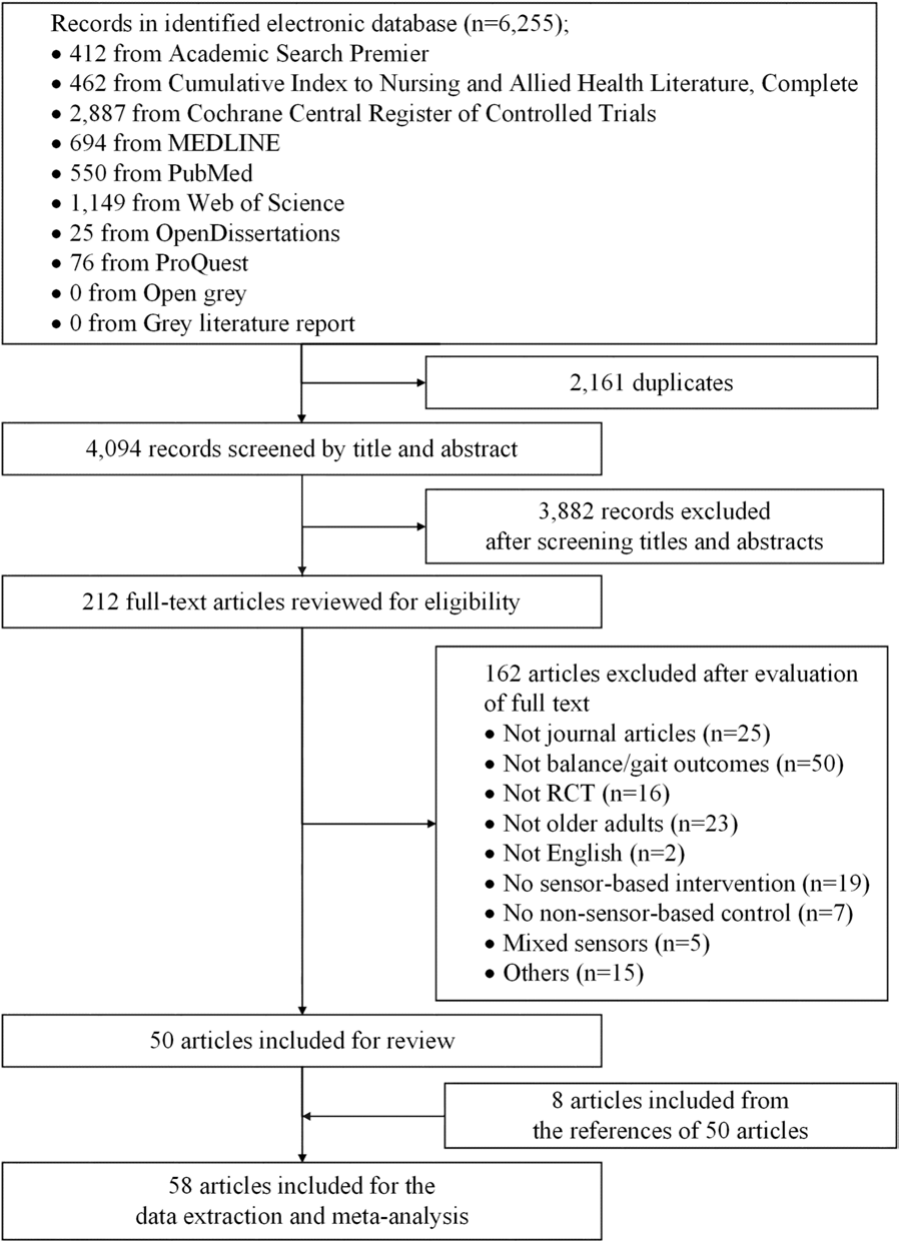
The flow of literature search and selection process

The literature search and review process is shown in Fig. 2. Of the 6255 articles identified from the ten databases, 2,161 were removed due to duplications. Two reviewers (Q.M. and J.Z.) screened the remaining 4094 articles by reading the titles and abstracts. Finally, the full text of 212 potential articles was further reviewed, and 60 articles met the inclusion criteria. However, 10 of the 60 studies did not provide sufficient data and the authors did not reply to our requests for data. After manually searching the reference lists of the remaining 50 articles, eight additional relevant studies were identified, leading to 58 studies eligible for inclusion. Therefore, 58 studies were included in the meta-analysis.

The detailed characteristics of the 58 included studies [17–19, 21, 23, 36–88] can be found in Appendix 1. All the included studies tested SI groups (OPTS, PCPS, or WS) and control groups (TPEI groups and/or NTI groups), including 2,713 older adults. The mean age ranged from 60.25 to 86.90 (standard deviation [SD] = 2.80 to 17.14) in the SI groups and from 60.20 to 87.50 (SD = 3.00 to 13.04) in the control groups. In the SI groups within 58 included studies, 27, 24, and 7 trials utilized OPTS, PCPS, and WS, respectively. Forty-eight control groups received TPEI (e.g., treadmill training, tai chi, balance training, traditional strengthening exercises, or gait training), while 26 control groups received NTI. The treatment sessions in each study ranged from 6 to 100 min, with 1–7 sessions per week. The entire training duration in these studies ranged from 1 to 26 weeks.

### Meta-analysis of the effects on outcomes

**Fig. 3:**
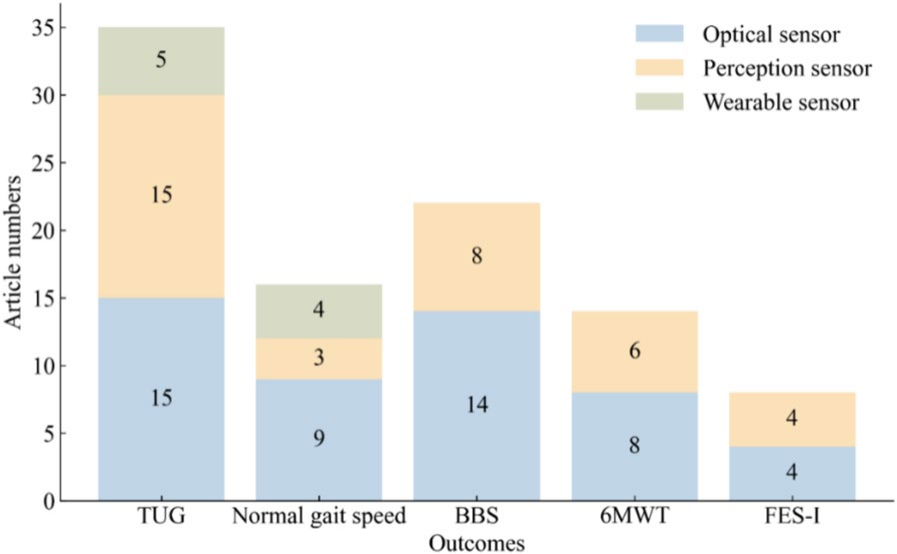
The number of trials using three sensors in the five outcomes

Of the 58 studies included in the review, we chose the following five gait and balance outcomes for our meta-analysis: the TUG test, normal gait speed, BBS, 6MWT, and FES-I scores. The sensor technologies included OPTS, PCPS, and WS. The number of articles using three sensors in five outcomes is shown in Fig. 3.

The characteristics of the SI groups in the included studies are shown in Table 1. Kinect (n = 22), undefined infrared sensors (n = 2), and image cameras (n = 3) were used as OPTS and provided whole-body motion data in the included studies. PCPS employed pressure boards (n = 19), mats (n = 3), and platforms (n = 3) to provide biofeedback of feet pressure. WS used smartphones (n = 2), inertial units (n = 2), or devices for immersive virtual reality (n = 3) to collect the motion of the partial body, such as head, hand, shank, thigh and lower back. Moreover, Kinect and Wii balance boards were the main devices for OPTS and PCPS groups, accounting for 81% and 76%, respectively. More information can be found in Appendix 1.

**Table 1:** The characteristics of the SI groups in the included studies

Sensor type	Devices	Article number	Biofeedback
Optical sensor	Kinect	22	Whole-body motion
	Undefined infrared sensors	2	Whole-body motion
	Web camera	1	Whole-body motion
	Smartphone camera	1	Whole-body motion
	BTS NIRVANA VR system	1	Whole-body motion
Perception sensor	Wii balance board	19	Feet pressure
	A step mat	1	Feet pressure
	Tymo system	1	Feet pressure
	A pressure-sensitive electronic mat	1	Feet pressure
	Biorescue platform	1	Feet pressure
	Impact dance platform	1	Feet pressure
	Dividat senso-step training platform	1	Feet pressure
Wearable sensor	Oculus VR headset and two controllers	1	The motion of head and hands
	HTC Vive headset and two controllers	1	The motion of head and hands
	VR glasses with smartphone	1	The motion of head
	Smartphone -accelerometers and gyroscopes	2	The motion of torso at the lower back
	Inertial sensors—a tri-axial accelerometer, gyroscope and magnetometer	2	The motion of shank, thigh and lower back

### TUG test

**Fig. 4:**
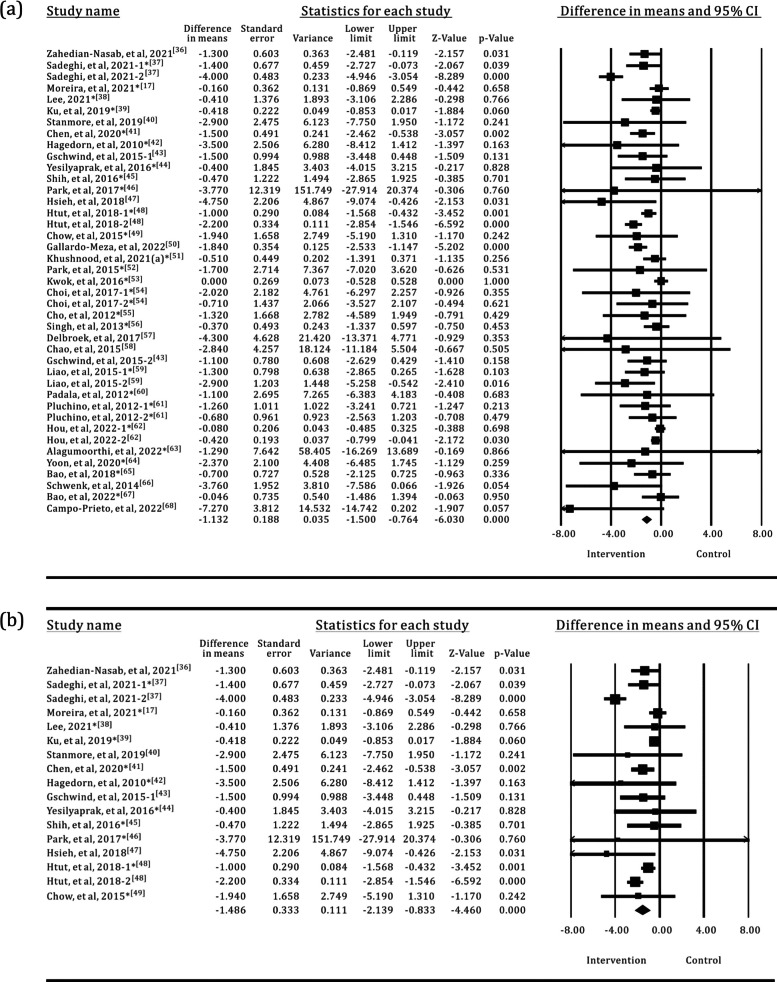

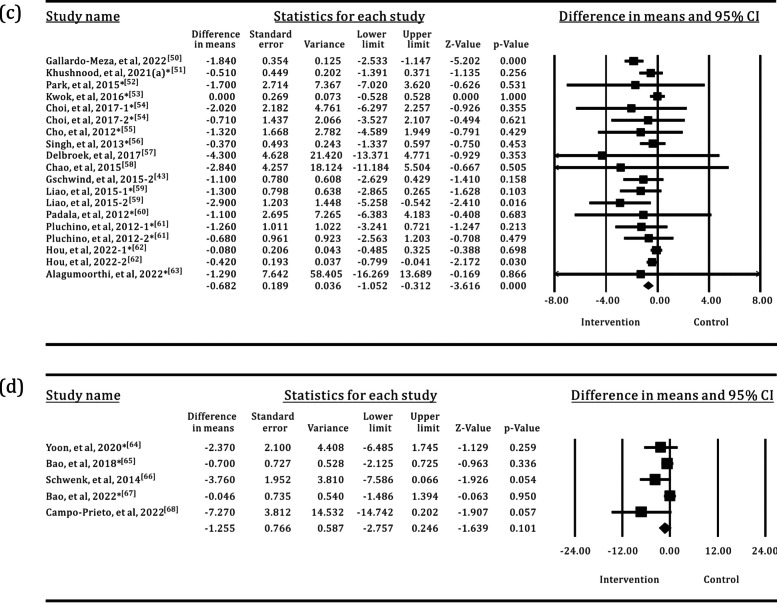
Forest plots for the effects of SI groups on TUG, as compared with control groups: **a** SI groups with all sensor technologies, **b** SI groups with OPTS, **c** SI groups with PCPS, **d** SI groups with WS

**Fig. 5:**
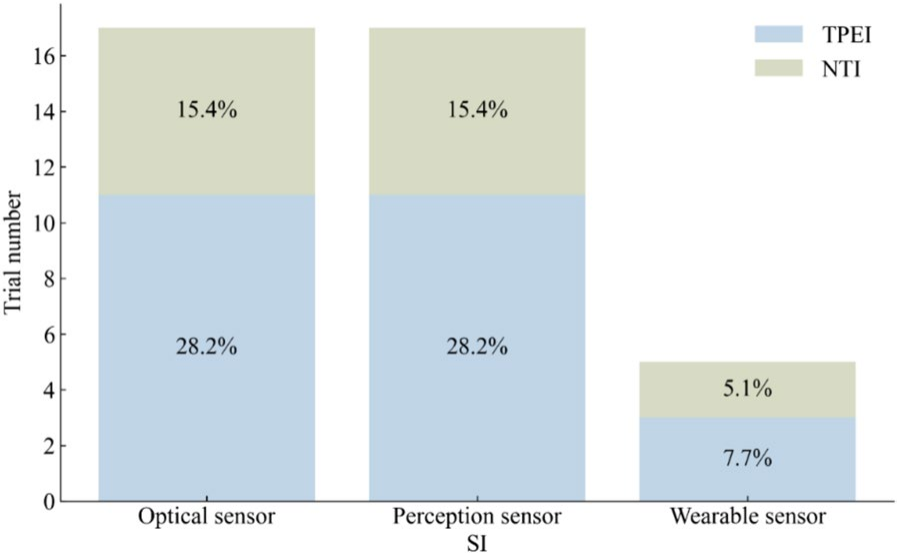
The interventions in meta-analyses for TUG

Of the 58 studies included in the analysis, 34 used the TUG test to assess the mobility of older adults, with a total of 750 participants in the SI groups and 875 participants in the control groups. The results of the meta-analysis are shown in Table 2 and Fig. 4. Most of the SI groups used OPTS or PCPS, and TPEI were used in approximately 64% of the control groups, as shown in Fig. 5. There were only five SI groups using WS to evaluate the TUG test performance. The results of the meta-analysis showed a statistically significant group difference between the SI and control or TPEI groups in the post-value of the TUG test. In the subgroup analysis of SI groups, statistically significant differences were also found between the OPTS or PCPS groups and control groups. Moreover, in the subgroup meta-analyses between specific SI and TPEI groups, statistically significant differences existed between the OPTS groups and TPEI groups; whereas no statistically significant difference was found between SI groups with PCPS and TPEI groups. Furthermore, there was no statistically significant difference between the WS groups and the control groups or the TPEI groups.

**Table 2:** Meta-analysis of the effects of SI versus control groups for TUG

Intervention group	No. of trials	I^2^	*p*(I^2^)	MD	95% CI	*p*
All group	34	67.851%	< 0.001	− 1.132	− 1.500, − 0.764	< 0.001
All group*	25	0.000%	0.609	− 0.448	− 0.641, − 0.255	< 0.001
OPTS	15	76.900%	< 0.001	− 1.486	− 2.139, − 0.833	< 0.001
OPTS*	11	5.700%	0.389	− 0.681	− 0.964, − 0.399	< 0.001
PCPS	15	40.524%	0.035	− 0.682	− 1.052, − 0.312	< 0.001
PCPS*	11	0.000%	0.894	− 0.226	− 0.499, 0.048	0.106
WS	5	41.281%	0.146	− 1.255	− 2.757, 0.246	0.101
WS*	3	0.000%	0.535	− 0.490	− 1.474, 0.493	0.328

*The control groups with TPEI only; TPEI: traditional physical exercise intervention; OPTS: optical sensor; PCPS: perception sensor; WS: wearable sensor; MD: mean difference

### Normal gait speed

**Fig. 6:**
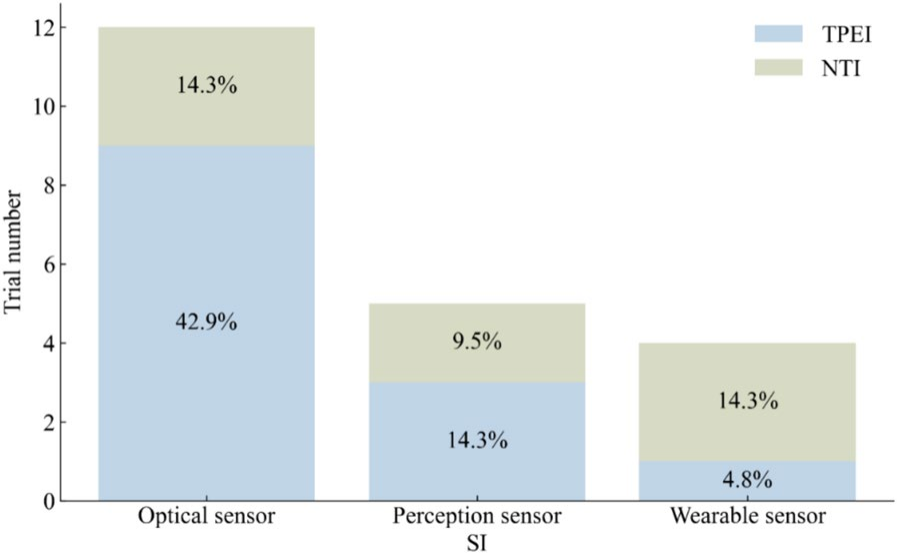
The interventions in meta-analyses for normal gait speed

**Fig. 7:**
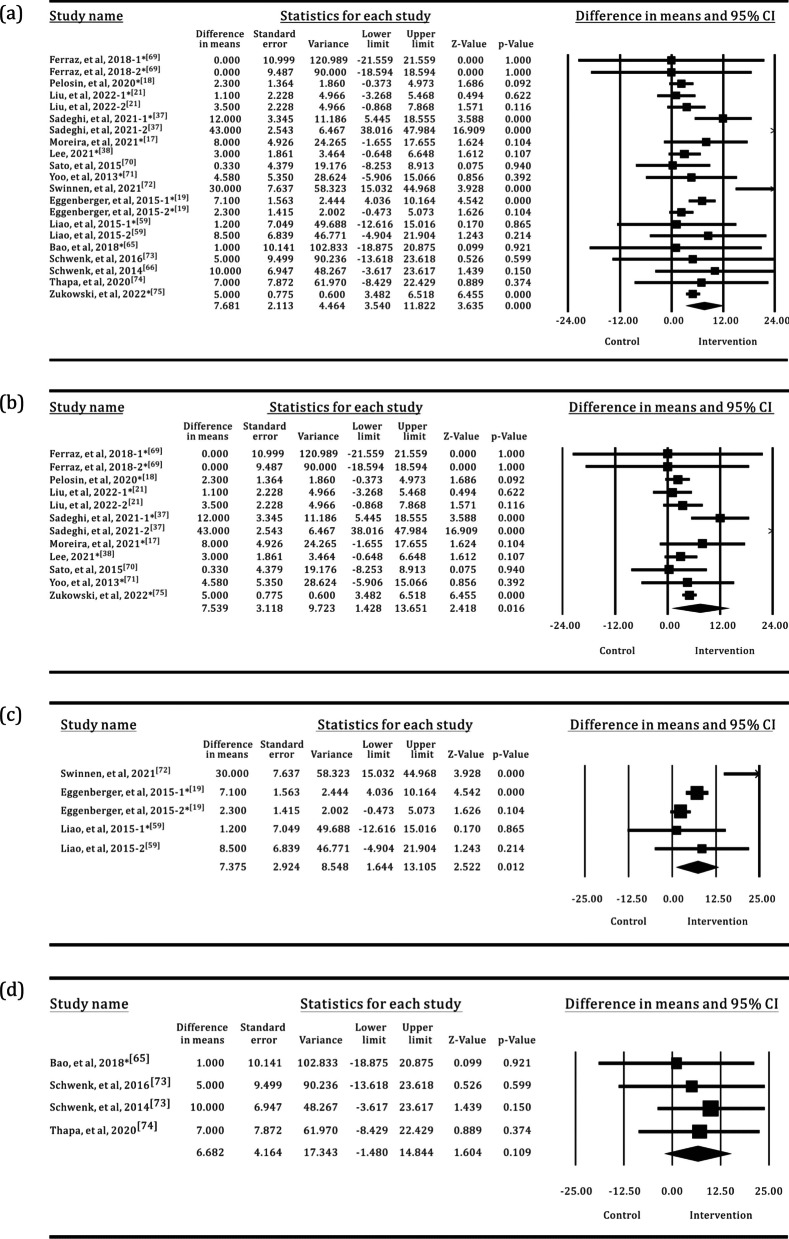
Forest plots for the effects of SI groups on normal gait speed, as compared with control groups: **a** SI groups with all sensor technologies, **b** SI groups with OPTS, **c** SI groups with PCPS, **d** SI groups with WS

Normal gait speed was assessed in 16 trials, with a total of 320 participants in the SI groups and 412 participants in the control groups. Almost half of the SI groups used OPTS, and 62% of the control groups used TPEI, as shown in Fig. 6. Three and four SI groups used PCPS and WS, respectively. The meta-analysis showed a statistically significant difference between the SI and the control groups or the TPEI groups, as shown in Table 3 and Fig. 7. When the SI groups were subdivided into three groups according to the sensor technology used, we also found a statistically significant difference between the OPTS or PCPS groups and the control groups or the TPEI groups. However, no statistically significant difference was found between WS groups and the control groups.

**Table 3:** Meta-analysis of the effects of SI versus control groups for normal gait speed

Intervention group	No. of trials	I^2^	*p*(I^2^)	MD	95% CI	*p*
All group	16	92.106%	< 0.001	7.681	3.540, 11.822	< 0.001
All group*	11	30.415%	0.141	4.272	3.268, 5.275	< 0.001
OPTS	9	95.324%	< 0.001	7.539	1.428, 13.651	0.016
OPTS*	8	31.821%	0.163	4.244	3.089, 5.399	< 0.001
PCPS	3	76.063%	0.002	7.375	1.644, 13.105	0.012
PCPS*	2	62.907%	0.067	4.382	0.321, 8.444	0.034
WS	4	0.000%	0.902	6.682	− 1.480, 14.844	0.109

*The control groups with TPEI only; TPEI: traditional physical exercise intervention; OPTS: optical sensor; PCPS: perception sensor; WS: wearable sensor; MD: mean difference

### BBS

**Fig. 8:**
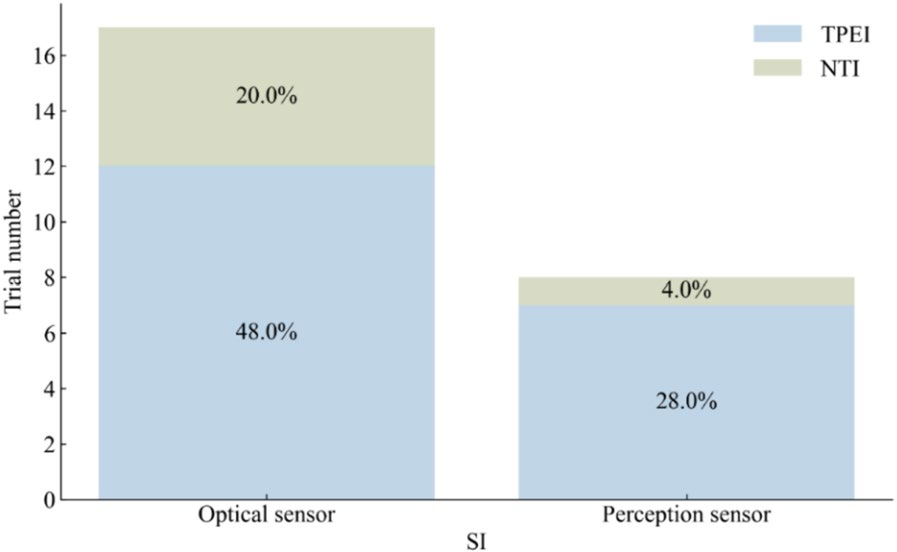
The interventions in meta-analyses for BBS

**Fig. 9:**
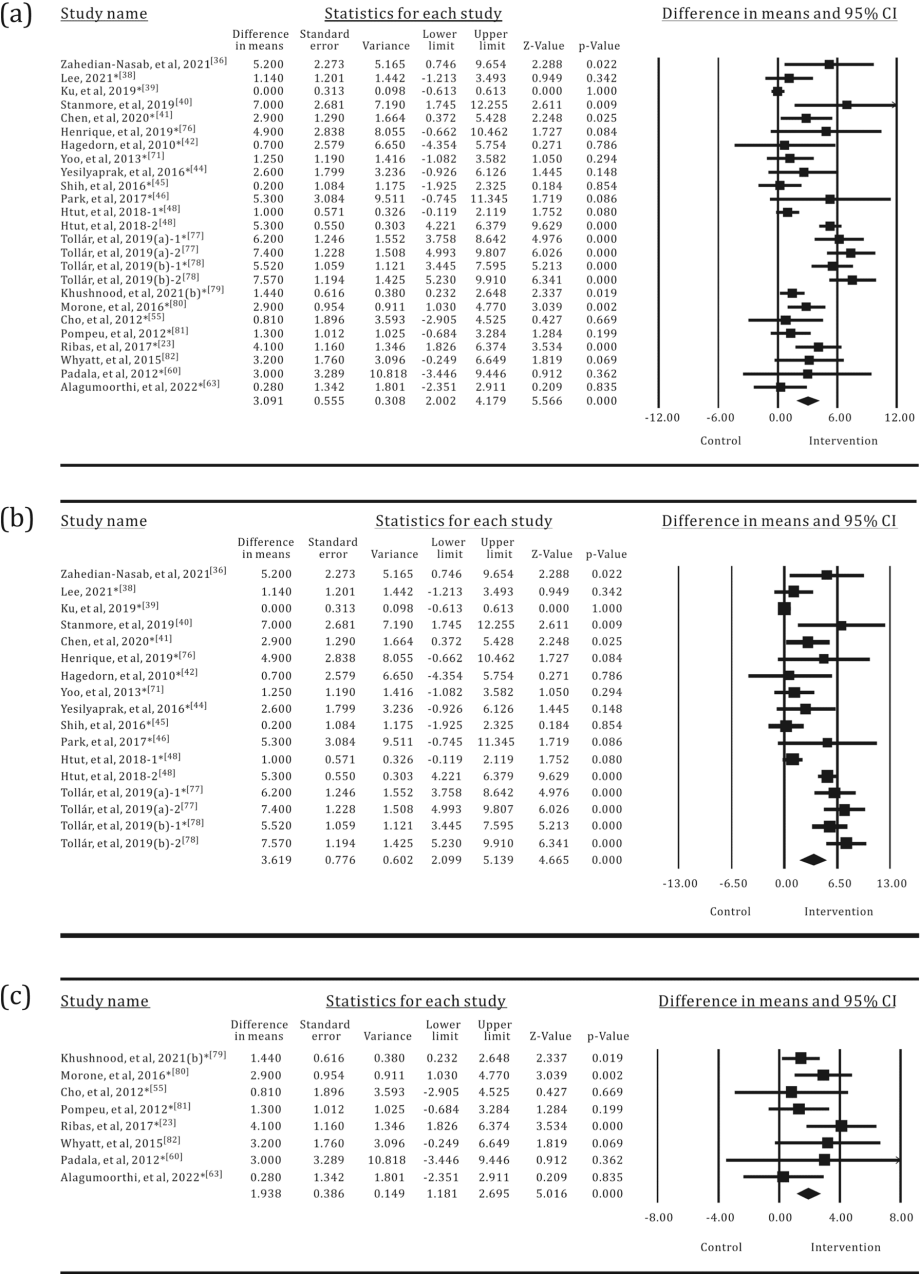
Forest plots for the effects of SI groups on BBS, as compared with control groups: **a** SI groups with all sensor technologies, **b** SI groups with OPTS, **c** SI groups with PCPS

The effectiveness of SI groups in improving postural balance was assessed using the BBS in 22 trials, with a total of 525 participants in the SI groups and 591 participants in the control groups. OPTS and PCPS were used in 64% and 36% of the SI groups. Approximately 76% of the control groups used TPEI, as shown in Fig. 8. The meta-analysis detected a statistically significant difference between the SI and the control groups or the TPEI groups, as shown in Table 4 and Fig. 9. In the meta-analysis of SI subgroups, statistically significant differences persisted between the OPTS or PCPS groups and the control groups or the TPEI groups.

**Table 4:** Meta-analysis of the effects of SI versus control groups for BBS

Intervention group	No. of trials	I^2^	*p*(I^2^)	MD	95% CI	*p*
All group	22	85.336%	< 0.001	3.091	2.002, 4.179	< 0.001
All group*	19	72.179%	< 0.001	2.133	1.213, 3.052	< 0.001
OPTS	14	89.717%	< 0.001	3.619	2.099, 5.139	< 0.001
OPTS*	12	79.343%	< 0.001	2.325	0.993, 3.657	0.001
PCPS	8	12.916%	0.329	1.938	1.181, 2.695	< 0.001
PCPS*	7	19.977%	0.277	1.874	1.098, 2.650	< 0.001

*The control groups with TPEI only; TPEI: traditional physical exercise intervention; OPTS: optical sensor; PCPS: perception sensor; MD: mean difference

### 6MWT

**Fig. 10:**
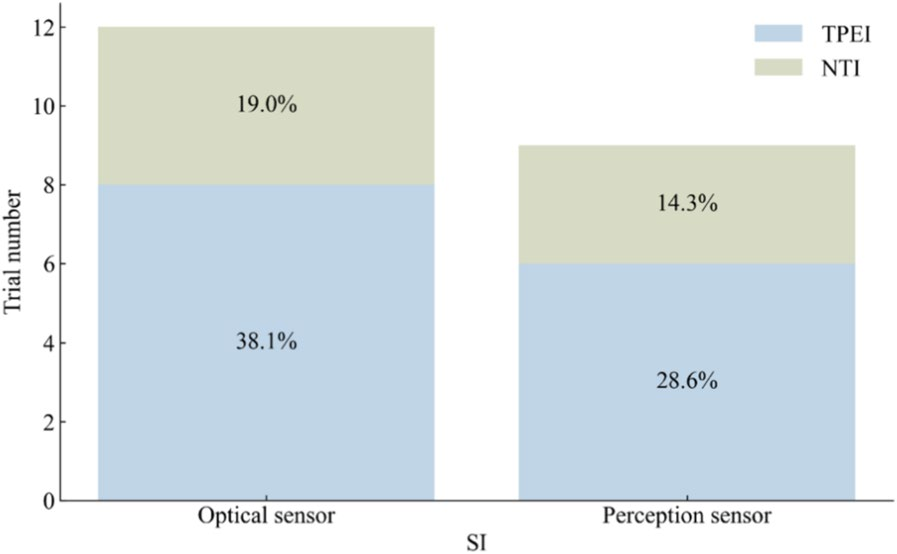
The interventions in meta-analyses for 6MWT

**Fig. 11:**
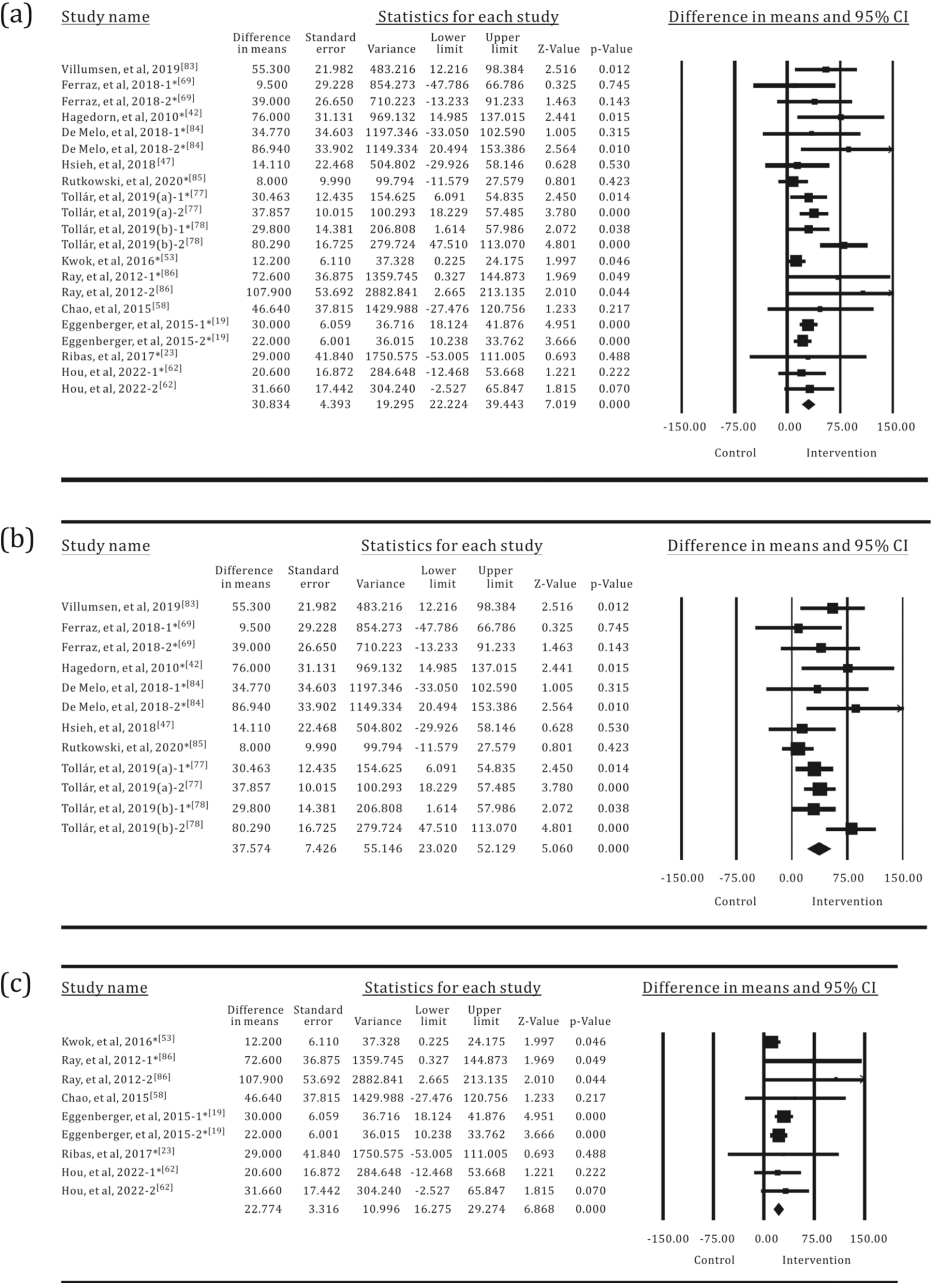
Forest plots for the effects of SI groups on 6MWT, as compared with control groups: **a** SI groups with all sensor technologies, **b** SI groups with OPTS, **c** SI groups with PCPS

The 6MWT was used to evaluate the walking ability of older adults in 14 trials, with a total of 323 participants in the SI groups and 473 participants in the control groups. OPTS and PCPS were used in approximately equal proportions in the SI groups, accounting for 57% and 43%, respectively. Approximately 66.7% of the control groups used TPEI, as shown in Fig. 10. The results of the meta-analysis are shown in Table 5 and Fig. 11. Statistically significant differences were found between the SI and the control groups or the TPEI groups. Furthermore, when the SI groups were classified into individual groups with the two sensor technologies, statistically significant differences were observed between the OPTS or PCPS groups and the control groups or the TPEI groups.

**Table 5:** Meta-analysis of the effects of SI versus control groups for 6MWT

Intervention group	No. of trials	I^2^	*p*(I^2^)	MD	95% CI	*p*
All group	14	41.657%	0.024	30.834	22.224, 39.443	< 0.001
All group*	11	20.287%	0.233	22.671	16.847, 28.495	< 0.001
OPTS	8	48.723%	0.029	37.574	23.020, 52.129	< 0.001
OPTS*	6	28.995%	0.197	25.166	13.158, 37.175	< 0.001
PCPS	6	15.524%	0.304	22.774	16.275, 29.274	< 0.001
PCPS*	5	19.782%	0.284	21.904	15.244, 28.563	< 0.001

*The control groups with TPEI only; TPEI: traditional physical exercise intervention; OPTS: optical sensor; PCPS: perception sensor; MD: mean difference

### FES-I

**Fig. 12:**
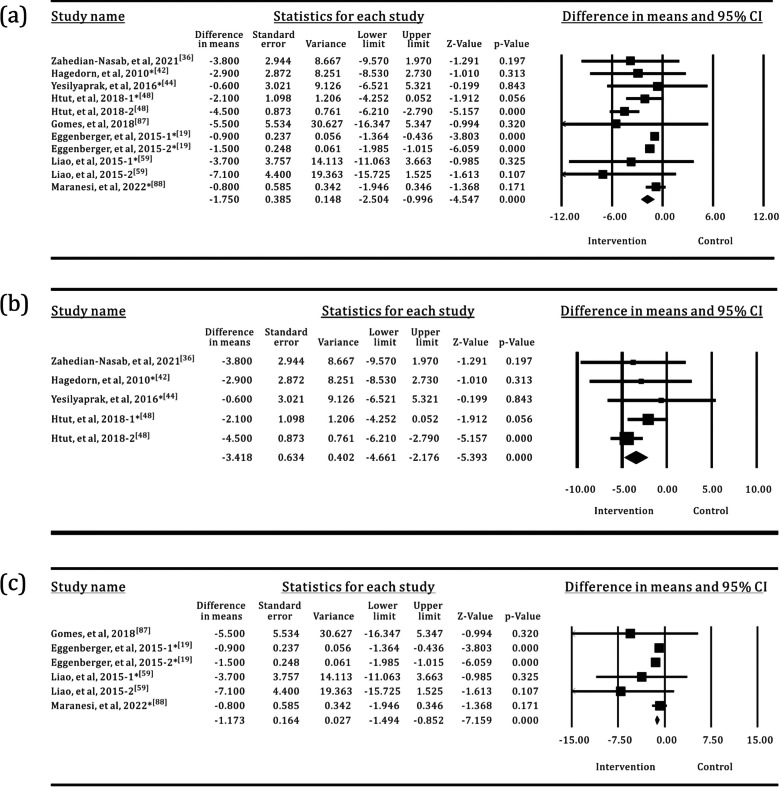
Forest plots for the effects of SI groups on FES-I, as compared with control groups: **a** SI groups with all sensor technologies, **b** SI groups with OPTS, **c** SI groups with PCPS

**Fig. 13:**
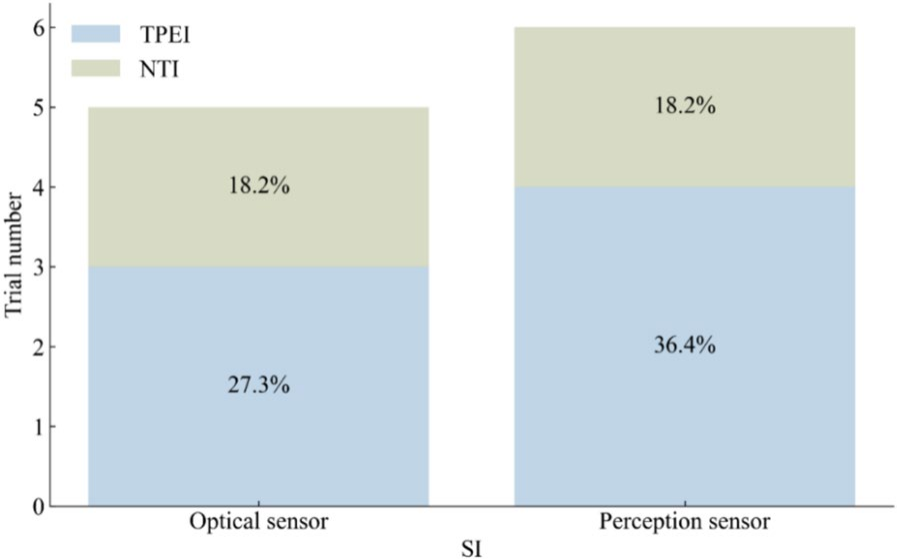
The interventions in meta-analyses for FES-I

Eight trials used the FES-I to measure the fear of falling in older adults. The sample size was 136 in the SI groups versus 194 in the control groups. The results of the meta-analysis are shown in Table 6 and Fig. 12. OPTS and PCPS were used in equal proportions (50%) in the SI groups, and none of the SI groups used WS. Approximately 63.7% of the control groups used TPEI, as shown in Fig. 13. The meta-analysis detected a statistically significant difference between the SI and the control groups or the TPEI groups. In the meta-analysis of SI subgroups, statistically significant differences were also identified between the OPTS or PCPS groups and the control groups or the TPEI groups.

**Table 6:** Meta-analysis of the effects of SI versus control groups for FES-I

Intervention group	No. of trials	I^2^	*p*(I^2^)	MD	95% CI	*p*
All group	8	54.587%	0.015	− 1.750	− 2.504, − 0.996	< 0.001
All group*	6	0.000%	0.539	− 1.185	− 1.502, − 0.868	< 0.001
OPTS	4	0.000%	0.420	− 3.418	− 4.661, − 2.176	< 0.001
OPTS*	3	0.000%	0.852	− 2.036	− 3.940, − 0.133	0.036
PCPS	4	21.390%	0.273	− 1.173	− 1.494, − 0.852	< 0.001
PCPS*	3	23.654%	0.269	− 1.161	− 1.482, − 0.839	< 0.001

*The control groups with TPEI only; TPEI: traditional physical exercise intervention; OPTS: optical sensor; PCPS: perception sensor; MD: mean difference

### Risk of bias

Of the 58 included trials, four articles using OPTS had a high risk of bias, 53 had some concerns, and one had a low risk of bias (see Appendix 2-Fig. 1). When removing the four articles with a high risk of bias, we reperformed the related meta-analyses to assess the effect of SI on gait or balance improvement (see Appendix 2-Table 1). We found that only one result changed from a statistically significant difference to no significant difference. Except for the meta-analysis comparing the normal gait speed in OPTS groups and control groups, the remaining meta-analyses still revealed that a statistically significant difference existed between SI groups and control or TPEI groups in terms of the outcomes for gait and balance evaluation.

### The quality of evidence

Based on the GRADE system, the evidence for effects on TUG test scores (SI groups with PCPS versus TPEI groups), BBS scores (SI groups with PCPS versus control or TPEI groups), 6MWT scores (SI groups with PCPS versus control or TPEI groups), and FES-I scores (SI groups with PCPS versus control groups) were evaluated as high quality. The evidence for effects on TUG test scores (SI groups with OPTS or WS versus TPEI groups; SI groups with PCPS versus control groups), normal gait speed (SI groups with PCPS or WS versus control groups; SI groups with OPTS versus TPEI groups), 6MWT scores (SI groups with OPTS versus TPEI groups), and FES-I scores (SI groups with OPTS versus control groups; SI groups with PCPS versus TPEI groups) was assessed as moderate quality. The evidence for effects on TUG test scores (SI groups with OPTS or WS versus control groups), normal gait speed (SI groups with OPTS versus control groups; SI groups with PCPS versus TPEI groups), BBS scores (SI groups with OPTS versus control groups or TPEI groups), 6MWT scores (SI groups with OPTS versus control groups), and FES-I scores (SI groups with OPTS versus TPEI groups) was assessed as low quality (see Appendix 3).

### Source of heterogeneity examination

In the TUG, BBS, 6MWT, and FES-I, the intervention strategy of control groups significantly contributed to a source of heterogeneity, as shown in Table 7. The meta-analyses of SI versus NTI groups achieved a larger effect size than that of SI versus TPEI groups. The participants’ age and health status also significantly affected the effect size of meta-analyses in terms of 6MWT. Based on the MD, young-old adults and individuals with Parkinson’s disease seemed to benefit more from the SI over other specific older adults. However, the sensor technology of SI groups showed no significant effect on the heterogeneity of meta-analyses among the five investigated outcomes (all *p* values > 0.05; see Appendix 4).

**Table 7:** Meta-regression and subgroup analyses for significant mediators of heterogeneity

	Mediators	No. of trials	I^2^	*p*(I^2^)	MD	95% CI	*p*(MD)	*p* (Meta-regression)
TUG	Intervention strategy of control groups							< 0.001
	TPEI	25	0.00%	0.609	− 0.448	− 0.641, − 0.255	< 0.001	
	NTI	14	81.61%	< 0.001	− 2.165	− 3.053, − 1.277	< 0.001	
BBS	Intervention strategy of control groups							< 0.001
	TPEI	19	72.18%	< 0.001	2.133	1.213, 3.052	< 0.001	
	NTI	6	30.27%	0.208	5.774	4.925, 6.624	< 0.001	
6MWT	Intervention strategy of control groups							0.049
	TPEI	11	20.29%	0.233	22.671	16.847, 28.495	< 0.001	
	NTI	7	33.54%	0.172	44.735	31.499, 57.970	< 0.001	
	Age							0.036
	Young-old adults	9	50.50%	0.016	31.595	19.611, 43.580	< 0.001	
	Old-old adults	5	22.92%	0.254	27.723	19.708, 35.738	< 0.001	
	Health status							0.035
	Chronic obstructive pulmonary disease	1	n/a	1	8	− 11.579, 27.579	0.423	
	Cognitive impairment	1	n/a	1	14.11	− 29.926, 58.146	0.53	
	Frailty	2	75.27%	0.044	36.797	− 24.065, 97.66	0.236	
	Health	2	17.56%	0.303	33.906	11.829, 55.982	0.003	
	Mobility impairment	1	n/a	0.643	34.948	19.661, 50.235	< 0.001	
	Parkinson’s disease	4	32.01%	0.184	46.618	29.601, 63.635	< 0.001	
	Prostate cancer	1	n/a	1	55.3	12.216, 98.384	0.012	
	Uncertain	2	n/a	0.556	26.221	17.917, 34.526	< 0.001	
FES-I	Intervention strategy of control groups							0.021
	TPEI	6	0.00%	0.539	− 1.185	− 1.502, − 0.868	< 0.001	
	NTI	4	0.00%	0.933	− 4.557	− 6.150, − 2.964	< 0.001	

*TPEI* traditional physical exercise intervention, *NTI* non-treatment intervention, *MD* mean difference, *TUG* Timed Up and Go, *BBS* Berg Balance Scale, *6MWT* 6-Minute Walk Test, *FES-I* Falling Efficacy Scale-International, *n/a* not applicable

## Discussion

The findings of SI’s effectiveness in this study seem to apply to mixed populations, as the heterogeneity of participants’ age and health status had no statistical impact on TUG, normal gait speed, BBS, and FES-I results. However, we found that young-old adults and individuals with Parkinson’s disease were more likely to benefit from SI in improving 6MWT. In addition, even though the sensor technology of SI groups was not a statistically significant mediator of heterogeneity, OPTS, PCPS, and WS groups showed different effectiveness compared with TPEI groups. More detailed information on the effectiveness of specific SI was discussed in the following sections.

### Effectiveness of sensor-based technologies in improving gait and balance performance

This review and meta-analysis provide a comprehensive synthesis of the effectiveness of SI in improving gait and balance performance in older adults, with a detailed classification of sensor technologies and control interventions. The results revealed that SI groups are statistically better than TPEI at improving TUG test, normal gait speed, BBS, 6MWT, and FES-I scores, which is consistent with the results of previous studies [20, 26, 27, 89]. The subgroup meta-analyses indicated that SI groups with OPTS (e.g., image cameras, Kinect, and infrared sensors) statistically outperform TPEI in improving the TUG test, normal gait speed, BBS, 6MWT, and FES-I scores. The effectiveness of SI groups with OPTS has also been proven on gait improvement in cognitive dual-task conditions [21].

The results of this meta-analysis suggest that SI groups with PCPS had more beneficial effects than TPEI groups on normal gait speed, BBS, 6MWT, and FES-I scores for gait and balance improvement [90]. However, SI groups with PCPS were no more statistically effective than TPEI groups in improving TUG test scores. Although the subgroup analysis results showed that interventions with WS (e.g., inertial sensors, smartphones used for inertial sensors, and head-mounted VR systems) presented no more statistical difference with control interventions on TUG test performance and normal gait speed [25–27], the effectiveness of WS was still uncertain due to the limited included studies and the heterogeneity among participants. Therefore, we suggest that more RCTs be conducted to verify the effects of WS-based interventions on different gait and balance outcomes [66].

Despite a statistically significant difference between SI and TPEI groups for TUG and Normal gait speed, the effect size doesn’t reach the minimal clinically important difference (MCID) (see Table 8). However, the BBS and 6MWT reached the MCID for the meta-analysis between SI and TPEI groups. Furthermore, the meta-regression results suggested no heterogeneity among the three types of sensor technologies in the meta-analyses. Regarding clinical impact, both OPTS and PCPS demonstrated a similar trend. The effect size values suggested that both OPTS and PCPS exceeded the MCID for BBS and 6MWT but not for TUG and normal gait speed. Additionally, the effect sizes of WS for normal gait speed, BBS, 6MWT, and FES-I were unavailable due to the limited number of RCTs. Hence, these findings suggested that OPTS and PCPS exhibit similar effectiveness in improving gait and balance. Nevertheless, OPTS showed a larger effect size than PCPS and WS in TUG, BBS, 6MWT, and FES-I, which might indicate a potential better effectiveness of OPTS. The following sections will further discuss the characteristics of three sensor technologies, the potential reasons for their difference in effectiveness, and their application scenarios.

**Table 8:** Comparison of the effect size of SI versus TPEI with MCID values

Outcome	SI	OPTS	PCPS	WS	MCID
TUG (s)	− 0.5	− 0.7	− 0.2	− 0.5	1.6 [91]
Normal gait speed (cm/s)	4.3	4.2	4.4	n/a	5.0 [92]
BBS	2.2	2.4	1.9	n/a	1.9 [93]
6MWT (m)	22.7	25.2	21.9	n/a	20.0 [92]
FES-I	− 1.2	− 2.0	− 1.2	n/a	No report [94]

*SI* sensor-based intervention, *OPTS* optical sensor, *PCPS* perception sensor, *WS* wearable sensor, *TUG* Timed Up and Go, *BBS* Berg Balance Scale, *6MWT* 6-Minute Walk Test, *FES-I* Falling Efficacy Scale-International, *MCID* minimal clinically important difference, *n/a* not applicable

### Characteristics of SI groups

Compared with conventional physical exercise interventions, SI groups provide immediate biofeedback consisting of several modes and contents [25, 27]. In the studies included in this meta-analysis, OPTS mainly used depth, infrared, and image cameras to track whole-body movements via interactive body motion detection technology. As Kinect has been certified as a safe and effective device for clinical use [95], it was the most popular device in the included studies (81.5% of OPTS). Force board, mat, and platform were used to monitor the center of pressure (CoP) of the feet in included SI groups with PCPS. Force platforms are the typical custom-made interactive posturography systems for balance training [82]. Physiotherapists commonly designed a series of specific platform-based exercises to enhance older adults’ awareness of body position and body stability via real-time visual and auditory feedback of accurate CoP [96]. However, the specialist administration and analysis of force platforms limits its application on daily exercise [82]. Thus, the Wii balance board, a commercially interactive system, was the most used device in included SI groups with PCPS due to its portability and affordability. Similar to the OPTS, included studies mainly used WS to provide the motion biofeedback of partial body based on inertial sensors.

Even though the statistically effect difference of OPTS, PCPS, and WS on gait and balance improvement remains to be proven, we deduced that mixed populations achieve the most benefits from the OPTS, followed by PCPS and WS according to the effect size values. One potential reason is that differences in biofeedback can cause various effects on training [97]. For example, the CoP and the movement signal of the lower back better provide the postural location and further reduce body sway than the movement signal of the upper trunk [97].

As the force feedback from weight-shifting can help older adults enhance their stance symmetry and mass translation [98], PCPS-based interventions that involve force biofeedback have been shown to improve the balance of patients with hemiplegia, spinal cord injuries, and traumatic brain injuries [99, 100]. However, our meta-analysis revealed that SI groups with PCPS showed no more improvements in TUG test scores than TPEI groups, which is consistent with the lower effectiveness of force feedback in improving functional ability and gait speed [98]. Although the weight-shifting tasks were helpful in improving postural stability, there is a limited relationship with gait performance or higher-level mobility tasks.

The OPTS captured whole-body movements without space limitation, including movements of the head, upper and lower trunk. Biofeedback from the lower trunk has similar effectiveness as CoP feedback in improving balance performance [97]. Thus, the SI with OPTS were more effective than TPEI groups in improving gait and balance. However, SI groups with OPTS showed no statistically better effectiveness over control groups in improving normal gait speed after removing one article with a high risk of bias. One possible reason is that two included studies performed balance-oriented tasks to compare the effectiveness of SI group with OPTS over NTI group [21, 70]. As balance skills tend to transfer no gait performance [101], the SI group with OPTS presented no statistically positive effect on gait improvement. Therefore, although there is a correlation between gait and balance [14], it is better to design specific interventions for different aspects.

The WS in this meta-analysis also provided movement biofeedback, but not all WS in the included studies were located at the lower back position. As upper trunk movement feedback is not effective in decreasing lower trunk tilt or CoP motion [97], SI with WS showed no statistically significant improvement in gait or balance compared with control interventions in this study. Further studies may focus on the lower track biofeedback when designing the SI groups with WS.

The accuracy of biofeedback is another potential reason for the different effects of the three sensor technologies. The main OPTS component sensor, Kinect, has been shown to have good consistency with Vicon, the “gold standard for movement analysis”, at measuring trunk and lower-extremity kinematics [102]. Moreover, compared with the “gold standard for quantifying center of pressure” (i.e., the lab-grade force platform), the Wii balance board, which is the main PCPS technology, has also been shown to perform well in terms of validity (intraclass correlation coefficients: 0.77–0.89) and reliability (intraclass correlation coefficients: 0.66–0.94) for measurements of CoP path length [103]. Although WS have been shown to have good validity (Pearson correlation coefficients: 0.68–0.99) and reliability (intraclass correlation coefficients: 0.85–0.94) in walking tests, unpredictable vibrations and the misplacement of sensors may lead to artifacts and inaccurate measurements [104, 105]. Thus, compared with OPTS and PCPS, WS may give relatively less accurate biofeedback due to their location characteristics. The WS included in the meta-analysis were mostly worn on the upper torso using elastic straps. Although the elastic straps are adjustable and easy to use, they tend to move with users’ exercise and put uncomfortable pressure on users [104]. The displacement of WS can contribute to incorrect biofeedback of partial body motion, which impacts the effectiveness of sensor-based intervention.

### Future research and limitations

According to the meta-analyses of TUG test, normal gait speed, BBS, 6MWT, and FES-I scores, sensor-based technologies significantly improved the gait and balance of older adults. Meanwhile, sensor-based technologies have the advantages of preventing monotony and boredom, enhancing adherence to training, and facilitating accessibility and ease of use [106–108]. They may be an alternative therapy to traditional physical exercises for improving gait and balance in older adults. The effectiveness of OPTS and PCPS, but not WS, in improving TUG, BBS, 6MWT, and FES-I scores suggests that they have great potential for promoting gait and balance in older adults.

Gait or balance rehabilitation interventions are very context- or task-specific [98]; thus, healthcare professionals (e.g., clinicians and physiotherapists) are recommended to identify and choose the sensor technology that best matches the rehabilitation needs. For example, as OPTS allow participants to move in a larger space and provide feedback on whole-body motion, this technology is applicable for gait training, and task design should be specific to gait. The PCPS are more suitable for balance intervention due to their limited space and force detection. Although this meta-analysis showed no statistically significant effectiveness of WS on gait or balance improvement over control interventions, we speculated that WS could be meaningful for improving gait and balance performance if the WS is worn on the lower trunk [25]. Therefore, we suggest that more studies be performed to assess the potential of WS-based technology. Additionally, more detailed designs of sensor-based technologies, including feedback modes, task types, and frequencies, are recommended to explore suitable SI for specific gait and balance improvement purposes.

There are several limitations to this systematic review and meta-analysis. First, as there were insufficient RCTs, the effectiveness difference among OPTS, PCPS and WS in improving gait and balance performance remains uncertain. Further investigation is recommended to compare the effectiveness of these three sensor technologies and identify the clinical significance differences in effect sizes. Second, it was difficult to investigate the differences between SI and TPEI groups for all the outcomes due to the limited number of RCTs. We recommend that more RCTs be conducted to compare the effectiveness of SI and TPEI in improving gait and balance performance and to explore the potential implications of sensor-based technologies. Moreover, the included studies reported no information regarding older adults’ technological familiarity and acceptance, which seems affect the effectiveness of SI on gait and balance improvements. Thus, the effectiveness of SI on gait and balance improvement may lack generalizability in specific groups and the optimal SI design remains unclear. Future meta-analyses should consider the abovementioned information and should be designed when sufficient public data are available.

## Conclusions

In this study, we performed a systematic review and meta-analysis to examine the effects of sensor-based technologies on gait and balance improvement among older adults. The results revealed that sensor-based interventions with biofeedback are statistically more effective than traditional physical exercises in improving older adults’ gait and balance performance, as determined by the TUG test, normal gait speed, BBS, 6MWT, and FES-I scores. In the subgroup meta-analyses of SI, we divided the sensor technologies into OPTS, PCPS, and WS. The results showed that OPTS with the biofeedback of whole-body motion or PCPS with the biofeedback of feet pressure were more effective than TPEI groups in improving gait and balance performance (except for TUG test scores for PCPS) in a mixed population of older adults. The OPTS are applicable for gait training, and PCPS are suited for balance interventions. However, participants’ age and health status potentially affected the effectiveness of SI on 6MWT. SI tended to present greater efficacy among young-old adults and individuals with Parkinson’s disease than other participants. We thus recommend further research to assess the effectiveness of SI on specific groups and consider the impact of intervention design.

### Supplementary Information

Supplementary Material 1.

Supplementary Material 2.

Supplementary Material 3.

Supplementary Material 4.

## Data Availability

The datasets generated and/or analyzed during the current study are available from the corresponding author on reasonable request.

## Abbreviations

SI: Sensor-based intervention
TPEI: Traditional physical exercise intervention
NTI: Non-treatment intervention
OPTS: Optical sensor
PCPS: Perception sensor
WS: Wearable sensor
TUG: Timed up and Go
BBS: Berg Balance Scale
6MWT: 6-Minute Walk Test
FES-I: Falling Efficacy Scale-International
RCTs: Randomized controlled trials
MCID: Minimal clinically important difference
