# Supplementary material for: Effectiveness of sensor-based interventions in improving gait and balance performance in older adults: systematic review and meta-analysis of randomized controlled trials
Source: J Neuroeng Rehabil. 2024 May 28;21:85. doi: 10.1186/s12984-024-01375-0 (PMC11131332; doi:10.1186/s12984-024-01375-0)
Supplement: Supplementary file 1 — Supplementary Material 1. [file 12984_2024_1375_MOESM1_ESM.docx]

**Appendix 1.** Characteristics of the 58 papers in the systematic review

| First author, publication year, study region | Sample size (N) and age (mean ± SD) (years) | Sensor type | Sensor-based interventions | Treatment in control groups | Trial duration  and frequency | Outcome measures |
| --- | --- | --- | --- | --- | --- | --- |
| Zahedian-Nasab, et al, 2021, Iran(36) | N= 60  SIG: N=30 (male =22);  age (69.67±7.725); CG: N=30 (male =22);  age (72±7.808); | Type: Kinect (OPTS) Amount: 1 Placement: Infront | Exercise by Xbox Kinect (penalty, goalkeeping, ski, and darts) | Routine programs of the nursing homes (jogging, table tennis, artistic activities) (TPEI) | 2 sessions per week, 30-60 min/ session, for 6 weeks | BBS; TUG; FES-I |
| Sadeghi, et al, 2021, Iran(37) | N= 44 SIG: N=15 (male=15);  age (74.1±7);  CG-1: N=14 (male =14);  age (70.4±4.3); CG-2: N=15 (male=15);  age (72.2±7.2); | Type: Kinect (OPTS) Amount: 1 Placement: Infront | VR exercise (the light race (stomp it) mini-game, the target kick, goalkeeper mini-games) | CG-1: Balance training (TPEI) CG-2: Non-treatment interventions (NTI) | 3 sessions per week, 40 min/ session, for 8 weeks | Normal gait speed; TUG |
| Moreira, et al, 2021, Brazil(17) | N=66 SIG: N=32 (male =0);  age (70.84±4.53); CG: N=34 (male =0);  age (70.76±5.60); | Type: Kinect (OPTS) Amount: 1 Placement: Infront | Exergaming: “your shape fitness evolved’’ game, strengthening exercises, balance and cardiorespiratory exercises | Traditional strengthening exercises, balance and cardiorespiratory exercises (TPEI) | 3 sessions per week, 50 min/ session, for 12 weeks | Normal gait speed; TUG |
| Lee, 2021, Korea(38) | N= 56 SIG: N=28 (male =16);  age (81.01±6.89);  CG: N=28 (male =15);  age (79.47±6.15); | Type: Smartphone-camera (OPTS) Amount: 1 Placement: Infront | Gait training with virtual reality | Gait training without virtual reality (TPEI) | 5 sessions per week, 50 min/ session, for 4 weeks | BBS; Normal gait speed; TUG |
| Ku, et al, 2019, Korea(39) | N= 34 SIG: N=18 (male =9);  age (64.7±7.27);  CG: N=16 (male =8);  age (65.0±4.77); | Type: Kinect (OPTS) Amount: 1 Placement: Infront | Novel three-dimensional interactive augmented reality system training ( the balloon game, cave game, and rhythm game) | Conventional physical fitness program - lower-extremity strengthening and endurance training thrice (TPEI) | 3 sessions per week, 30 min/ session, for 4 weeks | BBS; TUG |
| Stanmore, et al, 2019, UK(40) | N= 106 SIG: N= 56 (male =11);  age (77.9±8.9); CG: N= 50 (male =12);  age (77.8±10.2); | Type: Kinect (OPTS) Amount: 1 Placement: Infront | AGE UK staying steady falls prevention leaflet, Otago strength and balance home exercise leaflet, tailored exergame programme | AGE UK staying steady falls prevention leaflet, Otago strength and balance home exercise leaflet (NTI) | 3 sessions per week, tailored progression, for 12 weeks | BBS; TUG |
| Chen, et al, 2020, Taiwan(41) | N= 28 SIG: N= 14 (male =12);  age (72.2±2.8); CG: N= 14 (male =1);  age (75.1±5.5); | Type: Kinect (OPTS) Amount: 1 Placement: Infront | Augmented reality-assisted Tai-Chi training | Traditional Tai-Chi program (TPEI) | 3 sessions per week, 30 min/ session, for 8 weeks | BBS; TUG |
| Hagedorn, et al, 2010, Denmark(42) | N= 27  SIG: N= 15 (male =3);  age (81.5±7.7);  CG: N= 12 (female =6);  age (81.1±6.0); | Type: Infrared sensors (OPTS) Amount: 3 Placement: Around | Balance training with computer games | Traditional balance training (TPEI) | 2 sessions per week, 90 min/ session, for 12 weeks | 6MWT; TUG; BBS; FES-I |
| Gschwind, et al, 2015, Australia(43) | N= 124 SIG-1: N= 24 (male=9);  age (80.1±6.3); SIG-2: N= 39 (male=12);  age (82.5±7.0); CG: N= 61 (male=21);  age (80.2±6.5); | SIG-1: Type: Kinect (OPTS) Amount: 1 Placement: Infront SIG-2: Type: A pressure-sensitive electronic mat Amount: 1 Placement: Feet | SIG-1: Microsoft-Kinect exergames (balance exergames, strength exercises) SIG-2: Step-mat-training (a modified StepMania game, stepper, trail-stepping) | An educational booklet (NTI) | SIG-1: 180 min/ per week, for 16 weeks SIG-2: 3 sessions per week, 20 min/ session, for 16 weeks | TUG |
| Yeşilyaprak, et al, 2016, Turkey(44) | N= 18 SIG: N= 7 (male=4);  age (70.1±4.0); CG: N= 11 (male=2);  age (73.1±4.5); | Type: BTS NIRVANA VR system (OPTS) Amount: 1 Placement: Around | Balance training with the BTS NIRVANA VR interactive system | Conventional balance exercises (TPEI) | 3 sessions per week, 50~60 min/ session, for 6 weeks | BBS; TUG; FES-I |
| Shih, et al, 2016, Taiwan(45) | N=20 SIG: N=10 (male = 9);  age (67.5±9.96); CG: N=10 (male = 7);  age (68.8±9.67); | Type: Kinect (OPTS) Amount: 1 Placement: Infront | Balance-based exergaming | Conventional balance training (TPEI) | 2 sessions per week, 50 min/ session, for 8 weeks | BBS; TUG |
| Park, et al, 2017, South Korea(46) | N=20 SIG: N=10 (male = 5);  age (62±17.14); CG: N=10 (male = 5);  age (65.3±10.51); | Type: Kinect (OPTS) Amount: 1 Placement: Infront | VR-based exergaming and conventional physical therapy | Conventional physical therapy (TPEI) | SIG: 7 sessions per week, 60 min/ session, for 6 weeks CG: 7 sessions per week, 30 min/ session, for 6 weeks | BBS; TUG |
| Hsieh, et al, 2018, Taiwan(47) | N=60 SIG: N=31 (male = 7);  age (76.4±7.6); CG: N=29 (male = 10);  age (80±7.5); | Type: Kinect (OPTS) Amount: 1 Placement: Infront | Exergaming (Your shape fitness evolved 2012) | Non-treatment interventions (NTI) | 2 sessions per week, 60 min/ session, for 24 weeks | 6MWT; TUG |
| Htut, et al, 2018, Myanmar(48) | N= 63 SIG: N=21 (male =10);  age (75.8±4.89);  CG-1: N=21 (male =13);  age (75.9±5.65); CG-2: N=21 (male =12);  age (76.0±5.22); | Type: Kinect (OPTS) Amount: 1 Placement: Infront | Virtual reality-based exercise | CG-1: Physical exercise (TPEI) CG-2: Non-treatment interventions (NTI) | 3 sessions per week, 30 min/ session, for 8 weeks | BBS; TUG; FES-I |
| Chow, et al, 2015, Hong Kong(49) | N= 20 SIG: N=10 (male =3);  age (70.4±5.4); CG: N=10 (male =4);  age (68.0±3.0); | Type: Kinect (OPTS) Amount: 1 Placement: Infront | Cyber-golfing training | Regular table games (TPEI) | 7 sessions per week, 30~45 min/ session, for 2 weeks | TUG |
| Gallardo-Meza, et al, 2022, Chile(50) | N= 72 SIG: N=35 (male =0);  age (69.2±3.7); CG: N=37 (male = 0);  age (68.1±3.3); | Type: Wii balance board (PCPS) Amount: 1 Placement: Feet | Nintendo ‘‘Wii Fit Plus’’ gaming system ( sky slalom , sky jump , and balance bubble exergames) | Non-treatment interventions (NTI) | 2 sessions per week, 40 min/ session, for 4 weeks | TUG |
| Khushnood, et al, 2021(a), Pakistan(51) | N= 83 SIG: N=42 (male =26);  age (not given); CG: N=41 (male =25);  age (not given); | Type: Wii balance board (PCPS) Amount: 1 Placement: Feet | Game via Wii fit (Nintendo co.,ltd.)(basic step, basic run, soccer heading, table tilt, balance bubble and lotus focus) | Conventional balance training exercises - single-leg stand up, tandem walking, tip-toe walk, walk on heels, sideways walk, walking with high steps and contra-lateral arm raise (TPEI) | 2 sessions per week, 30 min/ session, for 8 weeks | TUG |
| Park, et al, 2015, Republic of Korea(52) | N= 24  SIG: N= 12 (male =9);  age (66.5±8.1);  CG: N= 12 (male =10);  age (65.2±7.9); | Type: Wii balance board (PCPS) Amount: 1 Placement: Feet | Wii fit balance exercise game/program: Soccer heading, snowboard slalom, and table tilt | Ball exercise (TPEI) | 3 sessions per week, 30 min/ session, for 8 weeks | TUG |
| Kwok, et al, 2016, Singapore(53) | N= 80  SIG: N= 40 (male =4);  age (70.5±6.7);  CG: N= 40 (male =8);  age (69.8±7.5); | Type: Wii balance board (PCPS) Amount: 1 Placement: Feet | Nintendo Wii Active gaming exercises (cardiovascular training, resistance band strengthening, calisthenics and balance training) | Traditional gym exercise - cardiovascular training, and balance and strength training (TPEI) | 1 session per week, 60 min/ session, for 12 weeks | TUG; 6MWT |
| Choi, et al, 2017, Republic of Korea(54) | N= 36 SIG-1: N= 12 (male =7);  age (61.25±5.59);  SIG-2: N= 12 (male =6);  age (62.58±5.51); CG: N= 12 (male =8);  age (61.92±6.08); | Type: Wii balance boards (PCPS) Amount: 2 Placement: Feet | SIG-1: Game-based constraint-induced movement therapy (ski slalom and soccer heading from the Wii fit game system: Two Wii balance boards) and traditional physical therapy SIG-2: General game-based training (one Wii balance boards without modifying the game) and traditional physical training | Traditional physical therapy - motor learning and neurodevelopmental treatment (TPEI) | 3 sessions per week, 30 min/ session, for 4 weeks | TUG |
| Cho, et al, 2012, Republic of Korea(55) | N= 22 SIG: N= 11 (male=8);  age (65.26±8.35); CG: N= 11 (female=6);  age (63.13±6.87); | Type: Wii balance board (PCPS) Amount: 1 Placement: Feet | Virtual reality balance training (balance bubble, ski slalom, ski jump, soccer heading, table tiling, and the penguin slide), standard rehabilitation program (physical therapy, occupational therapy, and speech-language therapy) | Standard rehabilitation program - physical therapy, occupational therapy, and speech-language therapy (TPEI) | SIG: 5 sessions per week, 60 min/ session, for 6 weeks; 3 sessions per week, 30 min/ session, for 6 weeks;  CG: 5 sessions per week, 60 min/ session, for 6 weeks | BBS; TUG |
| Singh, et al, 2013, Malaysia(56) | N=36 SIG: N= 18 (male=0);  age (61.12±3.72); CG: N= 18 (male=0);  age (64.00±5.88); | Type: Wii balance board (PCPS) Amount: 1 Placement: Feet | Virtual reality games (ski slalom, table tilt, penguin slide, soccer heading, tight rope walk, perfect 10 and tilt city) | Therapeutic balance exercise - one leg standing, free leg swinging, moving objects forward, backwards and sideways, and walking in place, sideways and backwards (TPEI) | 2 sessions per week, 40 min/ session, for 6 weeks | TUG |
| Delbroek, et al, 2017, Belgium(57) | N= 20 SIG: N= 10 (male=2);  age (86.9±5.6);  CG: N= 10 (male =5);  age (87.5±6.6); | Type: Biorescue -1,600 pressure sensors (PCPS) Amount: 1 Placement: Feet | A training program with the Biorescue | Non-treatment interventions (NTI) | 2 sessions per week, 18~30 min/ session, for 6 weeks | TUG |
| Chao, et al, 2015, USA(58) | N= 32 SIG: N= 16 (male=5);  age (86.63±4.18);  CG: N= 16 (male=3);  age (83.75±8.04); | Type: Wii balance board (PCPS) Amount: 1 Placement: Feet | SAHA program (motivational intervention and Wii fit exergames), a exercise-related poster and health education booklet | A exercise-related poster and health education booklet (NTI) | SIG: 2 sessions per week, 60 min/ session, for 4 weeks CG: 1 session per week, 30 min/ session, for 4 weeks | 6MWT; TUG |
| Liao, et al,2015, Taiwan(59) | N=36 SIG: N=12 (male = 6);  age (67.3±7.1); CG-1: N=12 (male = 6);  age (65.1±6.7); CG-2: N=12 (male = 5);  age (64.6±8.6); | Type: Wii balance board (PCPS) Amount: 1 Placement: Feet | Virtual reality-based Wii Fit exercise (Yoga exercises, Strengthening exercises, Balance games) | CG-1: Traditional exercise - stretching exercise, strengthening exercise, balance exercise (TPEI) CG-2: Non-treatment interventions (NTI) | 2 sessions per week, 45min/ session, for 6 weeks | Gait speed; Stride length; TUG; FES-I |
| Padala, et al, 2012, USA(60) | N=22 SIG: N=11 (male = 3);  age (79.3±9.8); CG: N=11 (male = 3);  age (81.6±5.2); | Type: Wii balance board (PCPS) Amount: 1 Placement: Feet | Wii-Fit program | Walking (TPEI) | 5 sessions per week, 30 min/ session, for 8 weeks | BBS; TUG |
| Pluchino, et al, 2012, USA(61) | N=40 SIG: N=12 (male = 4);  age (70.72±8.46);  CG-1: N=14 (male = 5);  age (76±7.74); CG-2: N=14 (male = 0);  age (69.28±6.03); | Type: Wii balance board (PCPS) Amount: 1 Placement: Feet | Wii Fit balance program | CG-1: Standardized balance exercise program (TPEI) CG-2: Tai Chi program (TPEI) | 2 sessions per week, 60 min/ session, for 8 weeks | TUG |
| Hou, et al, 2022, China(62) | N= 66 SIG: N=23 (male =5);  age (67.04±3.78); CG-1: N=22 (male =2);  age (67.81±4.96); CG-2: N=21 (male =6 );  age (67.52±4.94); | Type: A step mat -pressure sensors (PCPS) Amount: 1 Placement: Feet | Perform a dance mat version of the open-source DDR game Stepmania | CG-1: Video game training (TPEI) CG-2: Non-treatment interventions (NTI) | 3 sessions per week, 30 min/ session, for 12 weeks | TUG; 6MWT |
| Alagumoorthi, et al, 2022, India(63) | N= 192 SIG: N=96 (male =51); age (69.7±10); CG: N=96 (male =63); age (68.5 ±9.8); | Type: Wii balance board (PCPS) Amount: 1 Placement: Feet | Wii sports-based strategy training | Traditional balance training (TPEI) | 3 sessions per week, 30–40 min/session, for 12 weeks | TUG BBS |
| Yoon, et al, 2020, South Korea(64) | N= 30 SIG: N=15 (male =10);  age (72.26±3.65);  CG: N=15 (male =0);  age (71.86±4.89); | Type: VR glasses with smartphone (WS) Amount: 1 Placement: Head | Rehabilitation training in full immersion virtual reality using VR glasses ("Basejump VR: Wingsuit"), exercise therapy, continuous passive motion therapy | Exercise therapy -sandbag weighing, non-weight bearing knee extensor exercises, cycling, continuous passive motion therapy (TPEI) | SIG: 5 sessions per week, 80 min/ session, for 2 weeks CG: 5 sessions per week, 60 min/ session, for 2 weeks | TUG |
| Bao, et al, 2018, USA(65) | N= 12 SIG: N= 6 (male =1);  age (76.2±5.5);  CG: N= 6 (male =3);  age (75.0±4.7); | Type: Smartphone -accelerometers and gyroscopes (WS) Amount: 1 Placement: Around the torso at the L4/L5 level | In-home balance training exercises with smart phone balance trainer and sensory augmentation | In-home balance training exercises with smart phone balance trainer and without sensory augmentation (TPEI) | 3 sessions per week, 45 min/ session, for 8 weeks | TUG; Normal gait speed |
| Schwenk, et al, 2014, USA(66) | N= 33 SIG: N= 17 (male=7);  age (84.3±7.3);  CG: N= 16 (male=5);  age (84.9±6.6); | Type: Inertial sensors - a tri-axial accelerometer, gyroscope and magnetometer (WS) Amount: 5 Placement: Shank, thigh and lower back | Balance training (ankle point-to-point reaching tasks, and virtual obstacle crossing tasks) | Non-treatment interventions (NTI) | 2 sessions per week, 45 min/ session, for 4 weeks | Normal gait speed; TUG |
| Bao, et al, 2022, USA(67) | N= 15 SIG: N=8 (male =2); age (75.8±5.2); CG: N=7 (male =3); age (75.0±4.3); | Type: Smartphone -accelerometers and gyroscopes (WS) Amount: 1 Placement: Around the torso at the L4/L5 level | In-home balance training with sensory augmentation | In-home balance training without sensory augmentation (TPEI) | 3 sessions per week, 45 min/ session, for 8 weeks | TUG |
| Campo-Prieto, et al, 2022, Spain(68) | N= 24 SIG: N=13 (male =2); age (85.08±8.48); CG: N=11 (male =1); age (84.82±8.10); | Type: HTC Vive headset and two controllers (WS) Amount: 1 Placement: Head and hands | Immersive virtual reality training and usual care program | Usual care program (NTI) | 3 sessions per week, 6 min/ session, for 10 weeks | TUG |
| Ferraz, et al, 2018, Brazil(69) | N= 62  SIG: N= 20 (male =10);  age (not given); CG-1: N= 22 (male=16);  age (71);  CG-2: N= 20 (male=11);  age (not given); | Type: Kinect (OPTS) Amount: 1 Placement: Infront | Exergames (Xbox 360 video game: River rush, reflex ridge, 20,000 leaks) | CG-1: Functional training (TPEI) CG-2: Bicycle exercise (TPEI) | 3 sessions per week, 50 min/ session, for 8 weeks | 6MWT; Normal gait speed |
| Pelosin, et al, 2020, Italy(18) | N= 39; SIG: N=17 (male = 6); age: (73.2±3.6); CG: N=22 (male = 7);  age: (71.9±4.1); | Type: Kinect (OPTS) Amount: 1 Placement: Infront | Treadmill training non-immersive VR training (real-life challenges consisting of obstacles, pathways, and distracters) | Treadmill training (TPEI) | 3 sessions per week, 45 min/ session, for 6 weeks | Normal gait speed |
| Liu, et al, 2022, Taiwan(21) | N= 50 SIG: N=16 (male =4);  age (74.6±6.1);  CG-1: N=17 (male =5);  age (73.2±6.3); CG-2: N=17 (male =6);  age (73.4±6.5); | Type: Kinect (OPTS) Amount: 1 Placement: Infront | Tai Chi training during VR exergaming | CG-1: Yang style Tai Chi (TPEI) CG-2: Non-treatment interventions (NTI) | 3 sessions per week, 50 min/ session, for 12 weeks | Normal gait speed |
| Sato, et al, 2015, Japan(70) | N= 54 SIG: N= 28 (male =6);  age (70.07±5.35);  CG: N= 26 (female =5);  age (68.50±5.47); | Type: Kinect (OPTS) Amount: 1 Placement: Infront | Exergame with a Kinect sensor (apple game, tightrope standing game, balloon popping game, one-leg standing game) | Non-treatment interventions (NTI) | 2~3 sessions per week, 40~60 min/ session, for 8~12 weeks | BBS; Normal gait speed |
| Yoo, et al, 2013, Republic of Korea(71) | N= 21 SIG: N= 10 (male=0);  age (72.90±3.41); CG: N= 11 (male=0);  age (75.64±5.57); | Type: Web camera (OPTS) Amount: 1 Placement: Infront | Reality-based Otago exercise | Otago exercise (TPEI) | 3 sessions per week, 60 min/ session, for 12 weeks | BBS; Normal gait speed |
| Zukowski,, et al, 2022, USA(75) | N= 60 SIG: N=30 (male =8); age (71.2±6.5); CG: N=30 (male =9); age (72.0±7.7); | Infrared sensors (OPTS) Amount: None Placement: Around | Semi-immersive virtual reality treadmill training | Conventional treadmill training (TPEI) | 1 session for 30 min | Normal gait speed |
| Swinnen, et al, 2021, Belgium(72) | N= 45 SIG: N=23 (male =5);  age (84.7±5.6); CG: N=22 (male =5);  age (85.3±6.5); | Type: Dividat senso-step training platform - pressure sensors (PCPS) Amount: 1 Placement: Feet | Exergaming training | Listen to favorite music (NTI) | 3 sessions per week, 15 min/ session, for 8 weeks | Normal gait speed |
| Eggenberger, et al, 2015, Switzerland(19) | N= 71 SIG: N= 24 (male=10);  age (77.3±6.3); CG-1: N= 22 (male=6);  age (78.5±5.1); CG-2: N= 25 (male=9);  age (80.8±4.7); | Type: Impact dance platforms - pressure sensors (PCPS) Amount: 1 Placement: Feet | Video game dancing, complementary strength and balance exercises | CG-1: Treadmill memory training, complementary strength and balance exercises (TPEI) CG-2: Treadmill walking, complementary strength and balance exercises (TPEI) | 2 sessions per week, 60 min/ session, for 26 weeks | 6MWT; Normal gait speed; FES-I |
| Schwenk, et al, 2016, USA(73) | N= 22 SIG: N= 12 (male=5);  age (77.8±6.9);  CG: N= 10 (male=5);  age (79.0±10.4); | Type: Inertial sensors - a tri-axial accelerometer, gyroscope and magnetometer (WS) Amount: 5 Placement: The upper and lower legs, lower back | Balance training (weight shifting and virtual obstacle crossing) | Non-treatment interventions (NTI) | 2 sessions per week, 45 min/ session, for 4 weeks | Normal gait speed |
| Thapa, et al, 2020, Korea(74) | N= 68 SIG: N=34 (male =6);  age (72.6±5.4); CG: N=34 (male =10);  age (72.7±5.6); | Type: Oculus VR headset and two controllers (WS) Amount: 1 Placement: Head and hands | Instruction regarding VR training (juice making, crow shooting, fireworks, love house) and eye stretching exercises | Educational program on general health care (NTI) | SIG: 3 sessions per week, 100 min/ session, for 8 weeks CG: 1 session per week, 30~50 min/ session, for 8 weeks | Normal gait speed |
| Henrique, et al, 2019, Brazil(76) | N= 31  SIG: N= 16 (male =7); age (76.19±10.09);  CG: N= 15 (male =7); age (76.20±10.41); | Type: Kinect (OPTS) Amount: 1 Placement: Infront | Exergame rehabilitation using Motion Rehab AVE 3D | Conventional physiotherapy -flexion exercises, shoulder abduction and adduction, abduction and horizontal shoulder adduction, elbow extension, wrist extension, knee flexion, hip flexion, and abduction (TPEI) | 2 sessions per week, 30 min/ session, for 12 weeks | BBS |
| Tollár, et al, 2019(a), Hungary(77) | N= 83 SIG: N= 28 (male =14);  age (69.2±2.80); CG-1: N= 27 (male=12);  age (70.2±4.08); CG-2: N= 28 (male=13);  age (69.5±3.67); | Type: Kinect (OPTS) Amount: 1 Placement: Infront | Three Xbox 360 modules (reflex ridge, space pop, just dance) | CG-1: A “spinning class” - ride a bicycle ergometer at target hr with music (TPEI) CG-2: Non-treatment interventions (NTI) | 5 sessions per week, 60 min/ session, for 5 weeks | BBS; 6MWT |
| Tollár, et al, 2019(b), Hungary(78) | N= 74 SIG: N= 25 (male =12);  age (70.0±4.69); CG-1: N= 25(male =11);  age (70.6±4.10); CG-2: N= 24 (male=13);  age (67.5±4.28); | Type: Kinect (OPTS) Amount: 1 Placement: Infront | Three visual feedback modules of the Xbox 360 core system (reflex ridge, space pop, just dance) | CG-1: A “spinning class” - sit on the seat of a bicycle ergometer and rode at 110–140 beats/min (TPEI) CG-2: Non-treatment interventions (NTI) | 5 sessions per week, 60 min/ session, for 5 weeks | BBS; 6MWT |
| Khushnood, et al, 2021(b), Pakistan(79) | N= 83 SIG: N=42 (male =26);  age (not given);  CG: N=41 (male =25);  age (not given); | Type: Wii balance board (PCPS) Amount: 1 Placement: Feet | Exergaming via Wii fit | Conventional balance training exercises - high stepping, walking sideways, tandem walk and walking with head-turns (TPEI) | 2 sessions per week, 30 min/ session, for 8 weeks | BBS |
| Morone, et al, 2016, Italy(80) | N= 38 SIG: N= 19 (male =0);  age (67.8±2.98);  CG: N= 19 (female =0);  age (70.05±4.93); | Type: Wii balance board (PCPS) Amount: 1 Placement: Feet | A balance training with a Wii fit supervised by a physiotherapist ( diaphragmatic breathing exercises, yoga exercises, exercises muscle strengthening and flexibility, balance exercises) | Conventional balance exercises - stretching exercises and flexibility, strengthening of the antigravity muscles, balance exercises, postural exercises (TPEI) | 2 sessions per week, 60 min/ session, for 8 weeks | BBS |
| Pompeu, et al, 2012, Brazil(81) | N= 32 SIG: N= 16 (not given);  age (68.6±8.0);  CG: N= 16 (not given);  age (66.2±8.3); | Type: Wii balance board (PCPS) Amount: 1 Placement: Feet | Global exercises, and Wii-based motor and cognitive training | Global exercises, and balance exercise therapy (TPEI) | 2 sessions per week, 60 min/ session, for 7 weeks | BBS |
| Ribas, et al, 2017, Brazil(23) | N= 20 SIG: N= 10 (male=4);  age (61.70±6.83); CG: N= 10 (male=4);  age (60.20±11.29); | Type: Wii balance board (PCPS) Amount: 1 Placement: Feet | Exergaming exercise (table tilt, tilt city, penguin slide, soccer heading, basic run, obstacle course and basic step) | Conventional exercise - warming, stretching and active exercises; resistance exercises for the limbs; and diagonal exercises for the trunk, neck and limbs (TPEI) | 2 sessions per week, 30 min/ session, for 12 weeks | BBS; 6MWT |
| Whyatt, et al, 2015, UK(82) | N=82 SIG: N=40 (male = 5);  age (77.18±6.59); CG: N=42 (male =20);  age (76.62±7.28); | Type: Wii balance board (PCPS) Amount: 1 Placement: Feet | A structured balance training program | Non-treatment interventions (NTI) | 2 sessions per week, 30 min/ session, for 5 weeks | BBS |
| Villumsen, et al, 2019, Denmark(83) | N= 46 SIG: N= 23 (no given);  age (67.6±4.6); CG: N= 23 (no given);  age (69.8±4.4); | Type: Kinect (OPTS) Amount: 1 Placement: Infront | Homebased exergaming (aerobic and strength exercise-your shape fitness evolved 2012, sport and adventure games) | Non-treatment interventions (NTI) | 3 sessions per week, 60 min/ session, for 12 weeks | 6MWT |
| De Melo, et al, 2018, Brazil(84) | N=37 SIG: N=12 (male = 11);  age (60.25±9.28); CG-1: N=13 (male = 12);  age (61±10.72); CG-2: N=12 (male = 5);  age (65.58±13.04); | Type: Kinect (OPTS) Amount: 1 Placement: Infront | Exergaming (Your Shape – Fitness Evolved 2012 – Run the World) | CG-1: Walk or run maintaining the HR within the target range (TPEI) CG-2: Conventional gait training (TPEI) | 3 sessions per week, 20 min/ session, for 4 weeks | 6MWT |
| Rutkowski, et al, 2020, Poland(85) | N= 68 SIG: N=34 (male =6);  age (60.4±4.2); CG: N=34 (male =18);  age (62.1±2.9); | Type: Kinect (OPTS) Amount: 1 Placement: Infront | Traditional pulmonary rehabilitation and VR games | Traditional pulmonary rehabilitation program and endurance exercise training (TPEI) | 5 sessions per week, 35~55 min/ session, for 2 weeks | 6MWT |
| Ray, et al, 2012, US(86) | N= 87 SIG: N= 29 (not given);  age (not given);  CG-1: N= 40 (not given);  age (not given);  CG-2: N= 18 (not given);  age (not given); | Type: Wii balance board (PCPS) Amount: 1 Placement: Feet | Exercises with a Wii-balance board and weighted vests | CG-1: Exercises with traditional senior fitness programs (TPEI) CG-2: Non-treatment interventions (NTI) | 3 sessions per week, 45 min/ session, for 15 weeks | 6MWT |
| Gomes, et al, 2018, Brazil(87) | N= 30  SIG: N= 15 (not given);  age (83±5.87);  CG: N= 15 (not given); age (85±6.19); | Type: Wii balance board (PCPS) Amount: 1 Placement: Feet | Nintendo Wii Fit Plus™ games (Table Tilt, Rhythm Parade, Obstacle Course, Single Leg Extension, Tilty City, Basic Step, Penguin, Heading Soccer, Basic Run, Torso Twist) | A booklet with information and illustrations outlining the benefits and risks of physical activity (NTI) | 2 sessions per week, 50 min/ session, for 7 weeks | FES-I |
| Maranesi, et al, 2022, Italy(88) | N= 30 SIG: N=16 (male =6); age (72.7 ± 6.3); CG: N=14 (male =9); age (75.5 ± 5.4); | Type: Tymo system (PCPS) Amount: 1 Placement: Feet | Traditional therapy and treatment of Tymo system | Traditional therapy (TPEI) | 2 sessions per week, 50 min/session, for 5 weeks | FES-I |
| Note: *: the control groups with TPEI only; TPEI: traditional physical exercise intervention; NTI: Non-treatment intervention; OPTS: optical sensor; PCPS: perception sensor; WS: wearable sensor; TUG: Timed Up and Go; BBS: Berg Balance Scale; 6MWT: 6-Minute Walk Test; FES-I: Falling Efficacy Scale-International | | | | | | |
