# Supplementary material for: Effectiveness of sensor-based interventions in improving gait and balance performance in older adults: systematic review and meta-analysis of randomized controlled trials
Source: J Neuroeng Rehabil. 2024 May 28;21:85. doi: 10.1186/s12984-024-01375-0 (PMC11131332; doi:10.1186/s12984-024-01375-0)
Supplement: Supplementary file 2 — Supplementary Material 2. [file 12984_2024_1375_MOESM2_ESM.docx]

**Appendix 2**.


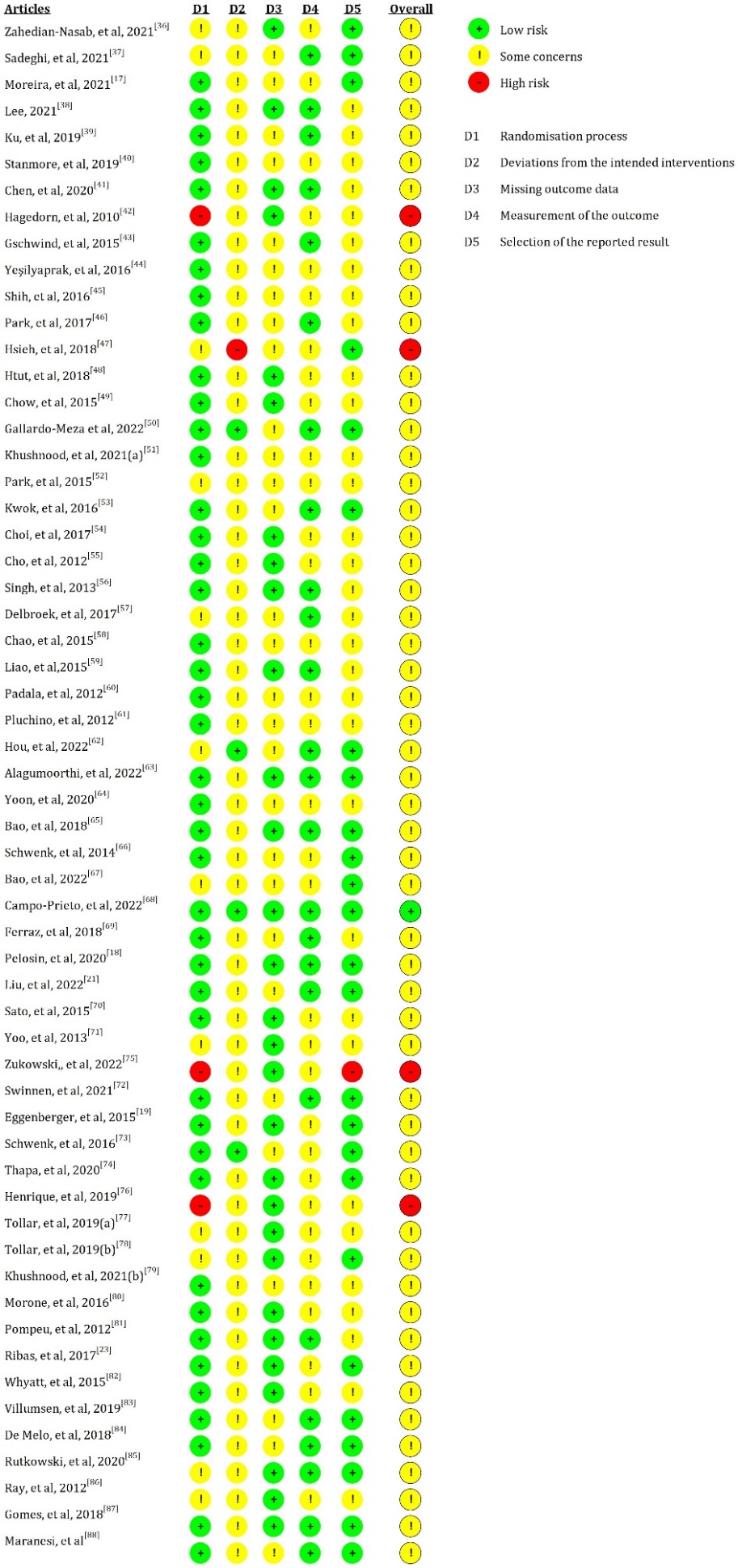


**Figure 1.** Assessment results of the risk of bias

**Table 1.** Meta-analysis of the effects of SI versus control groups for outcomes (excluding four articles with a high risk of bias)

| Intervention group | No. of trials | I^2^ | *p*(I^2^) | MD | 95% CI | *p* |
| --- | --- | --- | --- | --- | --- | --- |
| TUG |  |  |  |  |  |  |
| All group | 32 | 42.728% | 0.026 | 30.456 | 21.701, 39.210 | 0.000 |
| All group* | 24 | 10.091% | 0.344 | 22.181 | 16.330, 28.031 | 0.000 |
| OPTS | 13 | 52.234% | 0.027 | 37.584 | 21.972, 53.195 | 0.000 |
| OPTS* | 10 | 15.310% | 0.313 | 23.118 | 10.870, 35.366 | 0.000 |
| Normal Gait speed |  |  |  |  |  |  |
| All group | 15 | 92.457% | 0.000 | 7.815 | 2.787, 12.843 | 0.002 |
| All group* | 10 | 29.826% | 0.154 | 3.707 | 2.371, 5.043 | 0.000 |
| OPTS | 8 | 95.692% | 0.000 | 7.603 | -0.578, 15.785 | 0.069 |
| OPTS* | 7 | 26.103% | 0.220 | 3.205 | 1.425, 4.985 | 0.000 |
| BBS |  |  |  |  |  |  |
| All group | 20 | 86.451% | 0.000 | 3.113 | 1.990, 4.236 | 0.000 |
| All group* | 17 | 74.584% | 0.000 | 2.111 | 1.162, 3.059 | 0.000 |
| OPTS | 12 | 90.927% | 0.000 | 3.701 | 2.101, 5.302 | 0.000 |
| OPTS* | 10 | 82.457% | 0.000 | 2.299 | 0.889, 3.708 | 0.001 |
| 6MWT |  |  |  |  |  |  |
| All group | 12 | 42.728% | 0.026 | 30.456 | 21.701, 39.210 | 0.000 |
| All group* | 10 | 10.091% | 0.344 | 22.181 | 16.330, 28.031 | 0.000 |
| OPTS | 6 | 52.234% | 0.027 | 37.584 | 21.972, 53.195 | 0.000 |
| OPTS* | 5 | 15.310% | 0.313 | 23.118 | 10.870, 35.366 | 0.000 |
| FES-I |  |  |  |  |  |  |
| All group | 7 | 58.552% | 0.010 | -1.744 | -2.521, -0.967 | 0.000 |
| All group* | 5 | 0.000% | 0.456 | -1.180 | -1.497, -0.862 | 0.000 |
| OPTS | 3 | 22.346% | 0.277 | -3.445 | -4.719, -2.171 | 0.000 |
| OPTS* | 2 | - | - | - | - | - |
| Note: *: the control groups with TPEI only; TPEI: traditional physical exercise intervention; OPTS: optical sensor; MD: mean difference | | | | | | |
