# Supplementary material for: Effectiveness of sensor-based interventions in improving gait and balance performance in older adults: systematic review and meta-analysis of randomized controlled trials
Source: J Neuroeng Rehabil. 2024 May 28;21:85. doi: 10.1186/s12984-024-01375-0 (PMC11131332; doi:10.1186/s12984-024-01375-0)
Supplement: Supplementary file 3 — Supplementary Material 3. [file 12984_2024_1375_MOESM3_ESM.docx]

**Appendix 3.** Results of the GRADE assessment for quality of evidence

| No. of trials for outcomes | No. of participants | | Effect size (Hedges's g) (95% CI) | Quality of evidence |
| --- | --- | --- | --- | --- |
|  | Intervention group | Control group |  |  |
| TUG (n = 34) | 750 | 875 | -0.472 (-0.618, -0.327) | ⨁⨁◯◯^a,b^  Low |
| TUG (n = 25)* | 491 | 495 | -0.266 (-0.388, -0.143) | ⨁⨁⨁◯^a^  moderate |
| TUG (n = 15)^1^ | 303 | 377 | -0.637 (-0.924, -0.350) | ⨁⨁◯◯^a,b^  low |
| TUG (n = 11)^1^* | 169 | 178 | -0.432 (-0.643, -0.221) | ⨁⨁⨁◯^a^  moderate |
| TUG (n = 15)^2^ | 390 | 444 | -0.304 (-0.435, -0.173) | ⨁⨁⨁◯^b^  moderate |
| TUG (n = 11)^2^* | 293 | 289 | -0.168 (-0.325, -0.010) | ⨁⨁⨁⨁  high |
| TUG (n = 5)^3^ | 57 | 54 | -0.506 (-0.872, -0.141) | ⨁⨁◯◯^b,c^  low |
| TUG (n = 3)^3*^ | 29 | 28 | -0.324 (-0.824, 0.176) | ⨁⨁⨁◯^c^  moderate |
| Normal gait speed (n = 16) | 320 | 412 | 0.615 (0.318, 0.911) | ⨁⨁◯◯^a, b^  low |
| Normal gait speed (n = 11)* | 210 | 263 | 0.529 (0.229, 0.829) | ⨁⨁⨁◯^a^  moderate |
| Normal gait speed (n = 9)^1^ | 196 | 256 | 0.730 (0.259, 1.202) | ⨁⨁◯◯^a, b^  low |
| Normal gait speed (n = 8)^1^* | 168 | 198 | 0.527 (0.159, 0.896) | ⨁⨁⨁◯^a,^  moderate |
| Normal gait speed (n = 3)^2^ | 59 | 93 | 0.724 (0.273, 1.175) | ⨁⨁⨁◯^b^  moderate |
| Normal gait speed (n = 2)^2^* | 36 | 59 | 0.634 (-0.065, 1.332) | ⨁⨁◯◯^b,c^  low |
| Normal gait speed (n = 4)^3^ | 65 | 63 | 0.268 (-0.071, 0.608) | ⨁⨁⨁◯^c^  moderate |
| BBS (n = 22) | 525 | 591 | 0.731 (0.486, 0.976) | ⨁⨁◯◯^a,b^ low |
| BBS (n = 19)* | 406 | 403 | 0.559 (0.335, 0.783) | ⨁⨁◯◯^a,b^ low |
| BBS (n = 14)^1^ | 281 | 346 | 0.841 (0.518, 1.164) | ⨁⨁◯◯^a,b^ low |
| BBS (n = 12)^1^* | 202 | 200 | 0.599 (0.312, 0.886) | ⨁⨁◯◯^a,b^ low |
| BBS (n = 8)^2^ | 244 | 245 | 0.462 (0.172, 0.752) | ⨁⨁⨁⨁  high |
| BBS (n = 7)^2^* | 204 | 203 | 0.494 (0.141, 0.846) | ⨁⨁⨁⨁  high |
| 6MWT (n = 14) | 323 | 473 | 0.595 (0.466, 0.723) | ⨁⨁◯◯^a,b^ low |
| 6MWT (n = 11)* | 256 | 318 | 0.555 (0.397, 0.713) | ⨁⨁⨁◯^a^  moderate |
| 6MWT (n = 8)^1^ | 185 | 264 | 0.602 (0.373, 0.830) | ⨁⨁◯◯^a,b^ low |
| 6MWT (n = 6)^1^* | 133 | 163 | 0.471 (0.254, 0.688) | ⨁⨁⨁◯^a^  moderate |
| 6MWT (n = 6)^2^ | 138 | 209 | 0.616 (0.422, 0.810) | ⨁⨁⨁⨁  high |
| 6MWT (n = 5)^2^* | 123 | 155 | 0.672 (0.334, 1.009) | ⨁⨁⨁⨁  high |
| FES-I (n = 8) | 136 | 194 | -0.717 (-1.035, -0.399) | ⨁⨁◯◯^a,b^  low |
| FES-I (n = 6)* | 91 | 116 | -0.724 (-1.135, -0.313) | ⨁⨁⨁◯^a^  moderate |
| FES-I (n = 4)^1^ | 69 | 94 | -0.612 (-1.098, -0.126) | ⨁⨁⨁◯^a^  moderate |
| FES-I (n = 3)^1^* | 39 | 43 | -0.424 (-0.852, 0.004) | ⨁⨁◯◯^a,c^  low |
| FES-I (n = 4)^2^ | 67 | 100 | -0.804 (-1.247, -0.361) | ⨁⨁⨁⨁  high |
| FES-I (n = 3)^2^* | 52 | 73 | -0.946 (-1.539, -0.353) | ⨁⨁⨁◯^c^  moderate |
| *Note*:  *: the control groups with TPEI only; TPEI: traditional physical exercise interventions; TUG: Timed Up and Go; BBS: Berg Balance Scale; 6MWT: 6-Minute Walk Test; FES-I: Falling Efficacy Scale-International  ^1^ The trials with OPT sensors; OPT: optical;  ^2^ The trials with PCP sensors; PCP: perception;  ^3^ The trials with WS; WS: wearable sensors;  a. Downgraded by one level for risk of bias;  b. Downgraded by one level for inconsistency (I^2^ value is larger than 40%);  c. Downgraded by one level for imprecision (fewer than 150 participants). | | | | |
