# Supplementary material for: Effectiveness of sensor-based interventions in improving gait and balance performance in older adults: systematic review and meta-analysis of randomized controlled trials
Source: J Neuroeng Rehabil. 2024 May 28;21:85. doi: 10.1186/s12984-024-01375-0 (PMC11131332; doi:10.1186/s12984-024-01375-0)
Supplement: Supplementary file 4 — Supplementary Material 4. [file 12984_2024_1375_MOESM4_ESM.docx]

**Appendix 4.** Meta-regression and subgroup analyses for 58 included studies

|  | **Mediators** | **No. of trials** | **I^2^** | ***p*(I^2^)** | **MD** | **95% CI** | ***p*(MD)** | ***p* (Meta-regression)** |
| --- | --- | --- | --- | --- | --- | --- | --- | --- |
| **TUG** | **Sensor technology of SI groups** |  |  |  |  |  |  | 0.067 |
|  | - OPTS | 15 | 76.914% | <0.001 | -1.486 | -2.139, -0.833 | <0.001 |  |
|  | - PCPS | 15 | 40.524% | 0.035 | -0.682 | -1.052, -0.312 | <0.001 |  |
|  | - WS | 5 | 41.281% | 0.146 | -1.255 | -2.757, 0.246 | 0.101 |  |
|  | **Intervention strategy of control groups** |  |  |  |  |  |  | <0.001 |
|  | - TPEI | 25 | 0.000% | 0.609 | -0.448 | -0.641, -0.255 | <0.001 |  |
|  | - NTI | 14 | 81.613% | <0.001 | -2.165 | -3.053, -1.277 | <0.001 |  |
|  | **Age** |  |  |  |  |  |  | 0.592 |
|  | - Young-old adults (>=60 and <75 years old) | 21 | 72.112 | <0.001 | -1.014 | -1.439, -0.588 | <0.001 |  |
|  | - Old-old adults (>=75 years old) | 14 | 31.308 | 0.119 | -1.404 | -1.769, -1.040 | <0.001 |  |
|  | **Health status** |  |  |  |  |  |  | 0.555 |
|  | - Cognitive impairment | 3 | 0.000% | 0.565 | -3.407 | -6.546, -0.268 | 0.033 |  |
|  | - Fall risk | 4 | 0.000% | 0.524 | -1.277 | -2.277, -0.276 | 0.012 |  |
|  | - Frailty | 3 | 0.000% | 0.368 | -0.082 | -0.504, 0.339 | 0.702 |  |
|  | - Health | 10 | 86.171 | 0.000 | -1.220 | -1.779, -0.660 | <0.001 |  |
|  | - Parkinson's disease | 3 | 0.000% | 0.546 | -1.487 | -2.628, -0.345 | 0.011 |  |
|  | - Stroke | 3 | 0.000% | 0.959 | -1.195 | -3.099, 0.708 | 0.218 |  |
|  | - Total knee replacement | 1 | n/a | 1.000 | -2.370 | -6.485, 1.745 | 0.259 |  |
|  | - Uncertain | 8 | 0.000% | 0.577 | -0.719 | -1.282, -0.157 | 0.012 |  |
| **Normal gait speed** | **Sensor technology of SI groups** |  |  |  |  |  |  | 0.497 |
|  | - OPTS | 9 | 95.324% | <0.001 | 7.539 | 1.428, 13.651 | 0.016 |  |
|  | - PCPS | 3 | 76.063% | 0.002 | 7.375 | 1.644, 13.105 | 0.012 |  |
|  | - WS | 4 | 0.000% | 0.902 | 6.682 | -1.480, 14.844 | 0.109 |  |
|  | **Intervention strategy of control groups** |  |  |  |  |  |  | 0.095 |
|  | - TPEI | 11 | 0.141% | 30.415 | 4.272 | 3.268, 5.275 | <0.001 |  |
|  | - NTI | 8 | 0.000% | 95.742 | 13.596 | -1.633, 28.825 | 0.080 |  |
|  | **Age** |  |  |  |  |  |  | 0.852 |
|  | - Young-old adults | 10 | 0.000% | 94.423 | 7.538 | 1.429, 13.646 | 0.016 |  |
|  | - Old-old adults | 6 | 0.007% | 66.156 | 6.012 | 1.945, 10.078 | 0.004 |  |
|  | **Health status** |  |  |  |  |  |  | 0.738 |
|  | - Cognitive impairment | 4 | 70.191% | 0.009 | 6.967 | -0.048, 13.981 | 0.052 |  |
|  | - Fall risk | 3 | 0.000% | 0.545 | 2.727 | 0.597, 4.857 | 0.012 |  |
|  | - Frailty | 1 | n/a | 1.000 | 8.000 | -1.655, 17.655 | 0.104 |  |
|  | - Health | 3 | 97.074% | <0.001 | 14.706 | -8.266, 37.678 | 0.210 |  |
|  | - Parkinson's disease | 2 | 0.000% | 0.831 | 3.382 | -4.562, 11.325 | 0.404 |  |
|  | - Uncertain | 3 | 43.977% | 0.148 | 4.759 | -2.778, 6.739 | <0.001 |  |
| **BBS** | **Sensor technology of SI groups** |  |  |  |  |  |  | 0.240 |
|  | - OPTS | 14 | 89.717% | <0.001 | 3.619 | 2.099, 5.139 | <0.001 |  |
|  | - PCPS | 8 | 12.916% | 0.329 | 1.938 | 1.181, 2.695 | <0.001 |  |
|  | **Intervention strategy of control groups** |  |  |  |  |  |  | <0.001 |
|  | - TPEI | 19 | 72.179% | <0.001 | 2.133 | 1.213, 3.052 | <0.001 |  |
|  | - NTI | 6 | 30.269% | 0.208 | 5.774 | 4.925, 6.624 | <0.001 |  |
|  | **Age** |  |  |  |  |  |  | 0.907 |
|  | - Young-old adults | 15 | 86.165% | <0.001 | 3.100 | 1.781, 4.419 | <0.001 |  |
|  | - Old-old adults | 7 | 80.313% | <0.001 | 3.082 | 1.023, 5.141 | 0.003 |  |
|  | **Health status** |  |  |  |  |  |  | 0.560 |
|  | - Bone loss condition | 1 | n/a | 1.000 | 2.900 | 1.030, 4.770 | 0.002 |  |
|  | - Cognitive impairment | 1 | n/a | 1.000 | 2.000 | -3.446, 9.446 | 0.362 |  |
|  | - Fall risk | 3 | 22.192% | 0.277 | 2.174 | 0.382, 3.966 | 0.017 |  |
|  | - Frailty | 1 | n/a | 1.000 | 0.700 | -4.354, 5.754 | 0.786 |  |
|  | - Health | 4 | 94.441% | <0.001 | 2.082 | -0.005, 4.169 | 0.051 |  |
|  | - Mobility impairment | 1 | n/a | 0.493 | 6.809 | 5.094, 8.523 | <0.001 |  |
|  | - Parkinson's disease | 5 | 85.593% | <0.001 | 3.167 | 0.779, 5.556 | 0.009 |  |
|  | - Stroke | 3 | 13.231% | 0.316 | 2.740 | -0.011, 5.491 | 0.051 |  |
|  | - Uncertain | 3 | 50.948% | 0.130 | 3.087 | 0.216, 5.957 | 0.035 |  |
| **6MWT** | **Sensor technology of SI groups** |  |  |  |  |  |  | 0.802 |
|  | - OPTS | 8 | 48.723% | 0.029 | 37.574 | 23.020, 52.129 | <0.001 |  |
|  | - PCPS | 6 | 15.524% | 0.304 | 22.774 | 16.275, 29.274 | <0.001 |  |
|  | **Intervention strategy of control groups** |  |  |  |  |  |  | 0.049 |
|  | - TPEI | 11 | 20.287% | 0.233 | 22.671 | 16.847, 28.495 | <0.001 |  |
|  | - NTI | 7 | 33.543% | 0.172 | 44.735 | 31.499, 57.970 | <0.001 |  |
|  | **Age** |  |  |  |  |  |  | 0.036 |
|  | - Young-old adults | 9 | 50.502% | 0.016 | 31.595 | 19.611, 43.580 | <0.001 |  |
|  | - Old-old adults | 5 | 22.918% | 0.254 | 27.723 | 19.708, 35.738 | <0.001 |  |
|  | **Health status** |  |  |  |  |  |  | 0.035 |
|  | - Chronic obstructive pulmonary disease | 1 | n/a | 1.000 | 8.000 | -11.579, 27.579 | 0.423 |  |
|  | - Cognitive impairment | 1 | n/a | 1.000 | 14.110 | -29.926, 58.146 | 0.530 |  |
|  | - Frailty | 2 | 75.274% | 0.044 | 36.797 | -24.065, 97.66 | 0.236 |  |
|  | - Health | 2 | 17.557% | 0.303 | 33.906 | 11.829, 55.982 | 0.003 |  |
|  | - Mobility impairment | 1 | n/a | 0.643 | 34.948 | 19.661, 50.235 | <0.001 |  |
|  | - Parkinson’s disease | 4 | 32.008% | 0.184 | 46.618 | 29.601, 63.635 | <0.001 |  |
|  | - Prostate cancer | 1 | n/a | 1.000 | 55.300 | 12.216, 98.384 | 0.012 |  |
|  | - Uncertain | 2 | n/a | 0.556 | 26.221 | 17.917, 34.526 | <0.001 |  |
| **FES-I** | **Sensor technology of SI groups** |  |  |  |  |  |  | 0.486 |
|  | - OPTS | 4 | 0.000% | 0.420 | -3.418 | -4.661, -2.176 | <0.001 |  |
|  | - PCPS | 4 | 21.390% | 0.273 | -1.173 | -1.494, -0.852 | <0.001 |  |
|  | **Intervention strategy of control groups** |  |  |  |  |  |  | 0.021 |
|  | - TPEI | 6 | 0.000% | 0.539 | -1.185 | -1.502, -0.868 | <0.001 |  |
|  | - NTI | 4 | 0.000% | 0.933 | -4.557 | -6.150, -2.964 | <0.001 |  |
|  | **Age** |  |  |  |  |  |  | 0.581 |
|  | - Young-old adults | 4 | 0.000% | 0.483 | -1.061 | -2.145, 0.022 | 0.055 |  |
|  | - Old-old adults | 4 | 72.720% | 0.003 | -1.949 | -2.906, -0.992 | <0.001 |  |
|  | **Health status** |  |  |  |  |  |  | 0.961 |
|  | - Fall risk | 2 | 0.000% | 0.448 | -2.241 | -6.374, 1.891 | 0.288 |  |
|  | - Frailty | 2 | 0.000% | 0.677 | -3.452 | -8.449, 1.545 | 0.176 |  |
|  | - Health | 1 | n/a | 0.087 | -3.393 | -5.738, -1.048 | 0.005 |  |
|  | - Parkinson's disease | 2 | 21.664% | 0.279 | -0.974 | -2.097, 0.149 | 0.089 |  |
|  | - Uncertain | 1 | n/a | 0.080 | -1.196 | -1.784, -0.608 | <0.001 |  |
| Note: TPEI: traditional physical exercise intervention; NTI: non-treatment intervention; MD: mean difference; OPTS: optical sensor; PCPS: perception sensor; WS: wearable sensor; TUG: Timed up and Go; BBS: Berg Balance Scale; 6MWT: 6-Minute Walk Test; FES-I: Falling Efficacy Scale-International; n/a: not applicable. | | | | | | | | |
